# Anti-osteopontin therapy leads to improved edema and infarct size in a murine model of ischemic stroke

**DOI:** 10.1038/s41598-022-25245-8

**Published:** 2022-12-03

**Authors:** Daniel Spitzer, Tim Puetz, Moritz Armbrust, Maika Dunst, Jadranka Macas, Florian Croll, Karl-Heinz Plate, Yvonne Reiss, Stefan Liebner, Patrick N. Harter, Sylvaine Guérit, Kavi Devraj

**Affiliations:** 1 Edinger Institute (Institute of Neurology), University Hospital, Goethe University, 60528 Frankfurt, Germany; 2 Department of Neurology, University Hospital, Goethe University, 60528 Frankfurt, Germany; 3 German Cancer Consortium (DKTK), Partner site Frankfurt/Mainz, 60528 Frankfurt, Germany; 4 German Cancer Research Center (DKFZ), 69120 Heidelberg, Germany; 5 Frankfurt Cancer Institute (FCI), University Hospital, Goethe University, 60528 Frankfurt, Germany; 6 German Center for Cardiovascular Research (DZHK), Partner Site Frankfurt/Mainz, 60528 Frankfurt, Germany; 7 Excellence Cluster Cardio Pulmonary System (CPI), Partner Site Frankfurt, 60528 Frankfurt, Germany; 8 Center for Personalized Translational Epilepsy Research (CePTER), 60528 Frankfurt, Germany

**Keywords:** Blood-brain barrier, Neuroscience, Diseases, Neurological disorders, Stroke

## Abstract

Ischemic stroke is a serious neurological disorder that is associated with dysregulation of the neurovascular unit (NVU) and impairment of the blood–brain barrier (BBB). Paradoxically, reperfusion therapies can aggravate NVU and BBB dysfunction, leading to deleterious consequences in addition to the obvious benefits. Using the recently established EPAM-ia method, we identified osteopontin as a target dysregulated in multiple NVU cell types and demonstrated that osteopontin targeting in the early acute phase post-transient middle cerebral artery occlusion (tMCAO) evolves protective effects. Here, we assessed the time course of osteopontin and CD44 receptor expression in NVU cells and examined cerebroprotective effects of osteopontin targeting in early and late acute phases of ischemic stroke. Expression analysis of osteopontin and CD44 receptor post-tMCAO indicated increased levels of both, from early to late acute phases, which was supported by their co-localization in NVU cells. Combined osteopontin targeting in early and late acute phases with anti-osteopontin antibody resulted in further improvement in BBB recovery and edema reduction compared to targeting only in the early acute phase comprising the reperfusion window. Combined targeting led to reduced infarct volumes, which was not observed for the single early acute phase targeting. The effects of the therapeutic antibody were confirmed both in vitro and in vivo in reducing osteopontin and CD44 expression. Osteopontin targeting at the NVU in early and late acute phases of ischemic stroke improves edema and infarct size in mice, suggesting anti-osteopontin therapy as promising adjunctive treatment to reperfusion therapy.

## Introduction

Stroke is one of the primary causes of neurological disability and death world-wide. The majority of stroke cases (> 85%) fall under ischemic stroke that is characterized by a dysfunction of the neurovascular unit (NVU) and break down of the blood–brain barrier (BBB) in the early phases^1–3^. The neurological sequelae that follow in the later phases of stroke are primarily a result of neuronal damage occurring in the acute phase post ischemic stroke. Treatment of acute ischemic stroke focuses on intravenous thrombolysis with tissue plasminogen activator (IV-tPA), significantly improving outcomes when administered within the approved therapeutic window (4.5 h post-stroke onset)^4^. More recently, randomized clinical trials demonstrated that following IV tPA with mechanical thrombectomy, particularly in patients with large vessel occlusion, can improve outcomes further^5,6^. Moreover, mechanical thrombectomy may be offered to eligible patients up to 24 h after stroke onset, expanding treatment options for acute ischemic stroke patients^7^.

Although pharmacological and mechanical recanalization are highly effective reperfusion therapies, reperfusion may further aggravate BBB breakdown that contributes to the development of vasogenic brain edema and hemorrhagic transformation^8^, both of which exacerbate cerebral injury^9^. In this context, adjunctive neuroprotective treatment to reperfusion therapy in the acute phases is needed^10,11^.

We have recently identified osteopontin as a novel therapeutic target in ischemic stroke. Utilizing our recently introduced EPAM-ia method^12^, we isolated major NVU cell-types post ischemic stroke from mice and subjected them to RNA sequencing and bioinformatic analyses. Using this dataset (https://bioinformatics.mpi-bn.mpg.de/SGD_Stroke; NCBI GEO ID GSE163752) and using UpSet analysis (visualized at: http://vcg.github.io/upset/ submitting the JSON file link—https://raw.githubusercontent.com/SGD2020/mcao/master/mcao.json in the data address bar), we identified osteopontin (Spp1 gene product) as a target regulated in all major NVU cell types. Osteopontin as a potential therapeutic target was confirmed in human stroke specimen from acute phases. As osteopontin was upregulated in multiple NVU cell types that co-regulate the BBB function, we hypothesized a critical role of osteopontin in BBB dysfunction in stroke, that was also suggested by earlier reports^12–14^. In our previous work, we therefore targeted osteopontin using an anti-osteopontin IgG in the early acute phase in a murine model of transient middle cerebral artery occlusion (tMCAO). In vitro rescue using anti-osteopontin IgG was performed on primary mouse brain endothelial cells (MBMEC) subjected to oxygen glucose deprivation (OGD) to mimic stroke in vitro. Upon anti-osteopontin therapy, we demonstrated improved cerebral edema and BBB recovery, which was associated with improved neurological outcome and survival^12^. However, we did not observe an improvement in infarct size. In the current study, we therefore elaborated on our previous therapeutic strategy using anti-osteopontin IgG. We first analyzed post stroke mouse brains immunohistochemically at different time points to obtain the time course of expression for osteopontin and its receptor CD44, that was also reported to be detrimental in ischemic stroke^15^. Based on which, we formulated an extended therapeutic strategy comprising 2 doses of anti-osteopontin IgG. The first dose at 4 h i.e. in the early acute phase was like our previous study, which was combined with an additional dose at 15 h post stroke i.e. in the late acute phase. Using this regimen, we now show further improvement in edema, BBB recovery, and importantly reduced infarct size, that was associated with improved neurological outcome. Mechanistically, we show the involvement of osteopontin-CD44 pathway in BBB dysfunction both in vivo and in vitro. We therefore propose anti-osteopontin antibody as a novel adjunctive therapeutic in acute ischemic stroke.

## Results

### Time course of osteopontin and CD44 receptor expression in NVU cells in acute ischemic stroke

**Figure 1:**
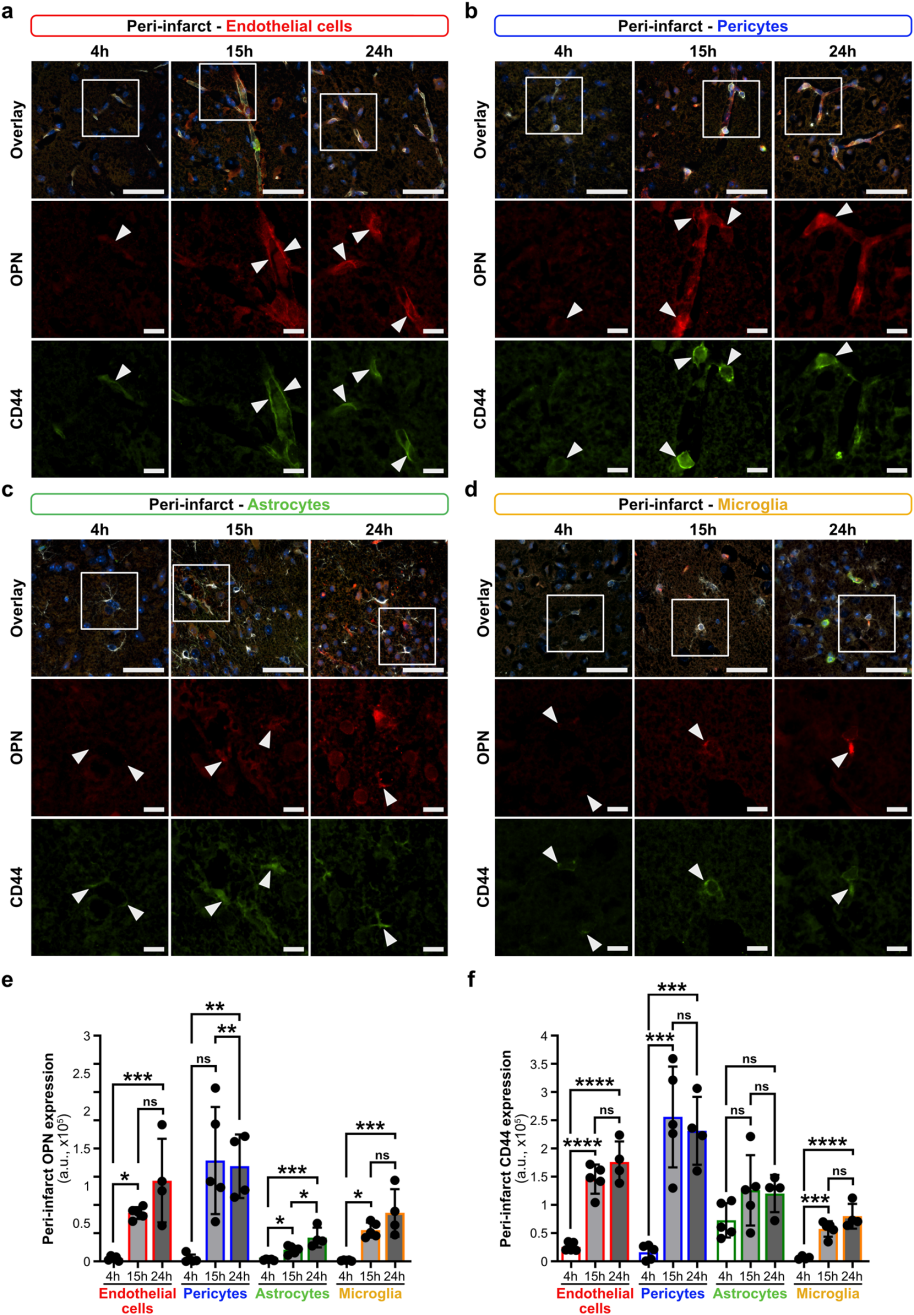
Time course of osteopontin and CD44 receptor expression in peri-infarct NVU cells in mice post-acute ischemic stroke. (**a**–**d**) Representative images of immunofluorescence staining for osteopontin (OPN, red, inset), CD44 receptor (green, inset) and cell-specific markers (white, overlay) including podocalyxin for endothelial cells (**a**), CD13 for pericytes (**b**), GFAP for astrocytes (**c**) and IBA1 for microglia/macrophages (**d**) in the peri-infarct region of mice 4 h (early acute phase), and 15 h and 24 h (late acute phase) post-stroke. White arrowheads indicate osteopontin and CD44 receptor expressing NVU cells. (**e**,**f**) Quantification of osteopontin and CD44 receptor expression intensity (arbitrary unit, a.u.) in peri-infarct NVU cells at indicated time points utilizing 3 images/region/animal, n = 5 (4 h), n = 5 (15 h) and n = 4 (24 h); *P < 0.05, **P < 0.01, ***P < 0.001, and not significant (ns) P > 0.05 by one-way ANOVA with Tukey's multiple comparisons test. Scale 50 µm, 10 µm in insets (**a**–**d**).

We showed previously that osteopontin is highly expressed in the extracellular space and in the major NVU cells (endothelial cells, pericytes, astrocytes and microglia) both in acute human and murine ischemic stroke lesions. We also showed that osteopontin unfolds deleterious effects on BBB function in a mouse model of acute ischemic stroke, further supporting brain edema formation and increasing the risk for hemorrhagic transformation^12^. CD44, a receptor for osteopontin^16^, which is a crucial osteopontin signaling pathway member, was also significantly upregulated in all major NVU cell types in acute murine ischemic stroke based on our recently published transcriptomic dataset^12^. Interestingly, CD44 has been reported to promote detrimental effects in cerebral ischemia^15^ and impairment of BBB integrity in vitro^17,18^. In view of the role of osteopontin and its receptor CD44 in stroke and BBB pathology, we first examined the time course of osteopontin and CD44 receptor expression in NVU cells in the peri-infarct region (Fig. 1) and infarct core (Supplementary Fig. S1) during the acute phase of ischemic stroke, including the early acute (4 h post-tMCAO) and late acute phase (15 and 24 h post-tMCAO). 4 h post-tMCAO, immunofluorescence staining revealed low expression of osteopontin and CD44 receptor in peri-infarct NVU cells that was comparable with the expression of OPN and CD44 receptor in NVU cells in the contralateral hemisphere (Supplementary Fig. S2). Interestingly, 15 and 24 h post-tMCAO a significant increase of osteopontin and CD44 receptor expression was observed in peri-infarct NVU cells, with the strongest increase 15 h post-stroke onset and most prominent in endothelial cells and pericytes (Fig. 1a,b,e,f and Supplementary Fig. S2). A similar time-dependent expression pattern was observed for co-localized osteopontin and CD44 receptor, suggesting ligand-receptor interaction. The co-localization of osteopontin and CD44 receptor was most prominent 15 h post-tMCAO in all the individual peri-infarct NVU cells (Supplementary Figs. S3 and S4), particularly in endothelial cells and pericytes. In the contralateral hemisphere, where osteopontin and CD44 receptor expression was low, osteopontin-CD44 receptor interaction was almost absent during the entire observation period (Supplementary Fig. S5). Moreover, the steep increase of osteopontin and CD44 receptor expression, and osteopontin-CD44 receptor interaction in peri-infarct glial cells (astrocytes and microglia/macrophages) 15 h post-onset of ischemic stroke (Fig. 1c,d, Supplementary Fig. S4) was associated with significantly increased expression of the glial activation markers GFAP in peri-infarct astrocytes and IBA1 in microglia/macrophages (Supplementary Fig. S4) compared to those of the contralateral hemisphere (Supplementary Fig. S5). A time-dependent increase of osteopontin and CD44 receptor expression (Supplementary Fig. S1), and osteopontin-CD44 receptor co-localization **(**Supplementary Figs. S3, S4) was also detected in NVU cells in the infarct core when compared to NVU cells in the contralateral hemisphere (Supplementary Figs. S2, S5). The only exception being microglia/macrophages, where the expression pattern was less prominent in the infarct core (Supplementary Figs. S2, S5). In this respect, immunofluorescence staining of stroke tissues revealed the most prominent increase of osteopontin and CD44 receptor expression, and osteopontin-CD44 co-localization in the infarct core for endothelial cells and microglia during the late acute phase of ischemic stroke. Thus, showing a significant increase of osteopontin expression and osteopontin-CD44 receptor interaction in endothelial cells 15 h post-tMCAO (Supplementary Figs. S1, S3), and osteopontin and CD44 expression and osteopontin-CD44 receptor co-localization in microglia/macrophages 15 and/or 24 h post-tMCAO (Supplementary Figs. S1, S4). For microglia/macrophages the increase of osteopontin and CD44 receptor expression and osteopontin-CD44 receptor co-localization was also associated with significantly elevated expression levels of the glial activation marker IBA1 15 h post-tMCAO (Supplementary Fig. S4).

### Anti-osteopontin antibody treatment 4 and 15 h post-tMCAO neutralizes osteopontin and suppresses CD44 receptor expression in NVU cells and improves neurological outcomes in mice

**Figure 2:**
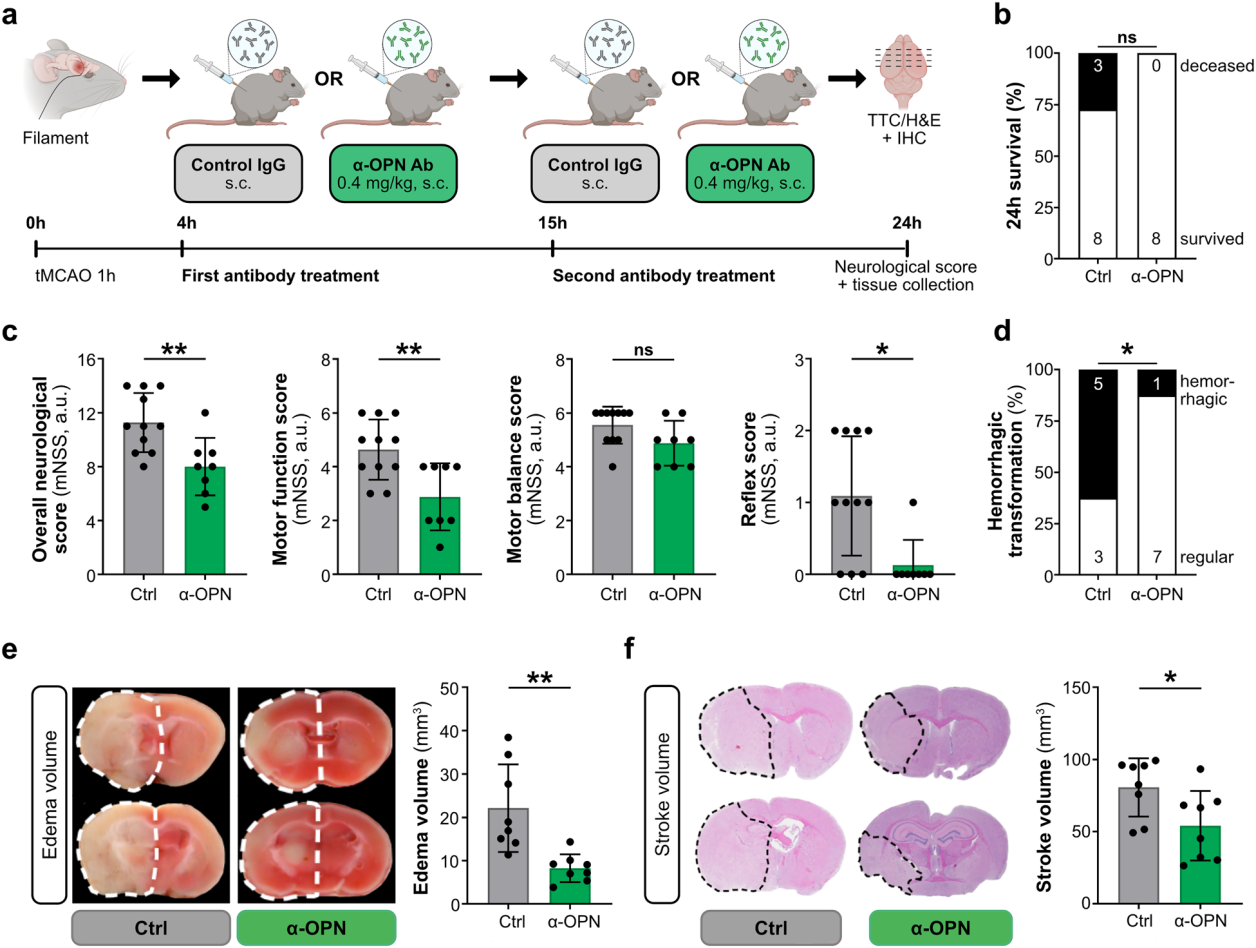
Neutralization of osteopontin during early and late acute phase of ischemic stroke improves neurological outcomes in mice. (**a**) Experimental timeline depicting subcutaneous (s.c.) administration of either 0.4 mg/kg control IgG or anti-osteopontin antibody (α-OPN) 4 h (early acute phase) and 15 h (late acute phase), and assessment of outcome parameters 24 h post-tMCAO. (**b**–**f**) Outcome parameters assessed 24 h after tMCAO in mice treated with Ctrl IgG or α-OPN antibody. All animals that passed the exclusion criteria were included for the survival and neurological score analysis. For analysis of hemorrhagic transformation, edema and stroke volumes only mice that survived 24 h were included. (**b**) 24 h survival proportion with numbers in histograms indicating animals that died or survived in each group, n = 11 (Ctrl) and n = 8 (α-OPN); not significant (ns) P > 0.05 by Chi-square test. (**c**) Total mNSS including motor balance, motor function and reflexes score, n = 11 (Ctrl) and n = 8 (α-OPN); *P < 0.05, **P < 0.01, and not significant (ns) P > 0.05 by Mann Whitney test. (**d**) Frequency of hemorrhagic transformation of stroke lesions. (**e**) Representative TTC-stained coronal brain slices of Ctrl IgG and α-OPN antibody-treated mice demonstrating edema-induced expansion of ischemic hemispheres as indicated by white dotted line (**e**, left panel), and corresponding edema volumes (**e** right panel). (**f**) Representative H&E-stained coronal brain slices of Ctrl IgG and α-OPN antibody-treated mice, demonstrating extent of infarction as indicated by black dotted line (**f**, left panel) and corresponding stroke volumes (**f**, right panel). n = 8 and 8 for Ctrl IgG and α-OPN antibody treatment group, respectively; *P < 0.05, **P < 0.01 by Chi-square test for (**d**), and two-tailed, unpaired t-test for (**e**,**f**).

**Figure 3:**
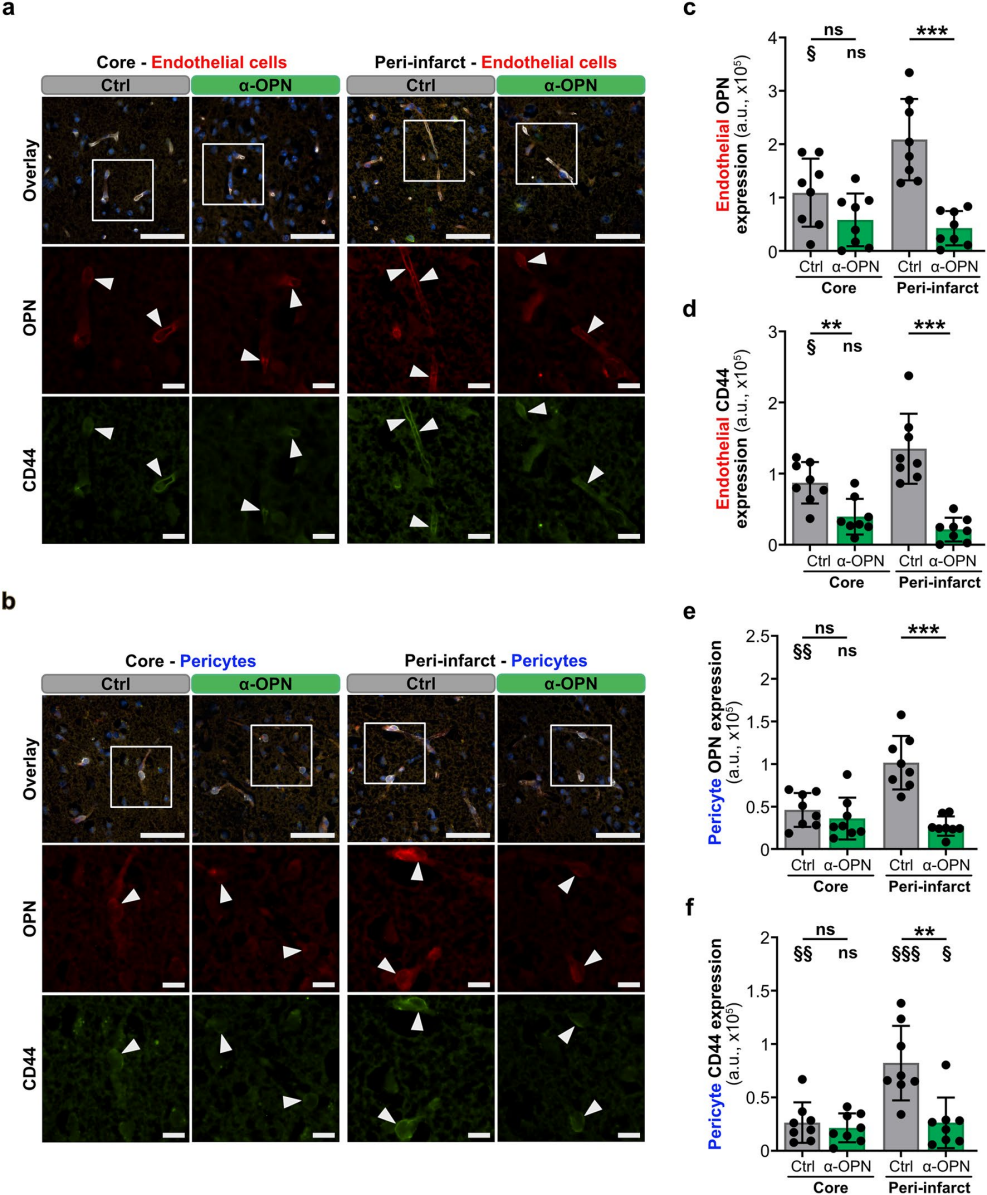
Anti-osteopontin antibody treatment reduces osteopontin and CD44 receptor expression in microvascular endothelial cells and pericytes after acute ischemic stroke. (**a**,**b**) Representative images of immunofluorescence staining for osteopontin (OPN, red, inset), CD44 receptor (green, inset) and cell-specific markers (white, overlay) including podocalyxin for endothelial cells (**a**) and CD13 for pericytes (**b**) in the infarct core and peri-infarct region of Ctrl IgG and α-OPN antibody-treated mice 24 h post-ischemic stroke. White arrowheads indicate osteopontin and CD44 receptor expressing endothelial cells and pericytes. (**c–f**) Quantification of osteopontin and CD44 receptor expression intensity (arbitrary unit, a.u.) in endothelial cells (**c**,**d**) and pericytes (**e**,**f**) in the infarct core and peri-infarct region utilizing 3 images/region/animal, n = 8 and 8 for Ctrl IgG and α-OPN antibody treatment group, respectively; ^§^P < 0.05, **/^§§^P < 0.01, ***P < 0.001 and not significant (ns) P > 0.05. *Indicates two-tailed, unpaired t-test with Welch’s correction when variances were significantly different based on F-test, comparing the two treatments groups for the same region, and § indicates two-tailed, paired t-test comparison of infarct core to peri-infarct region within the same treatment group. Scale bars 50 µm and 10 µm in insets (**a**,**b**).

**Figure 4:**
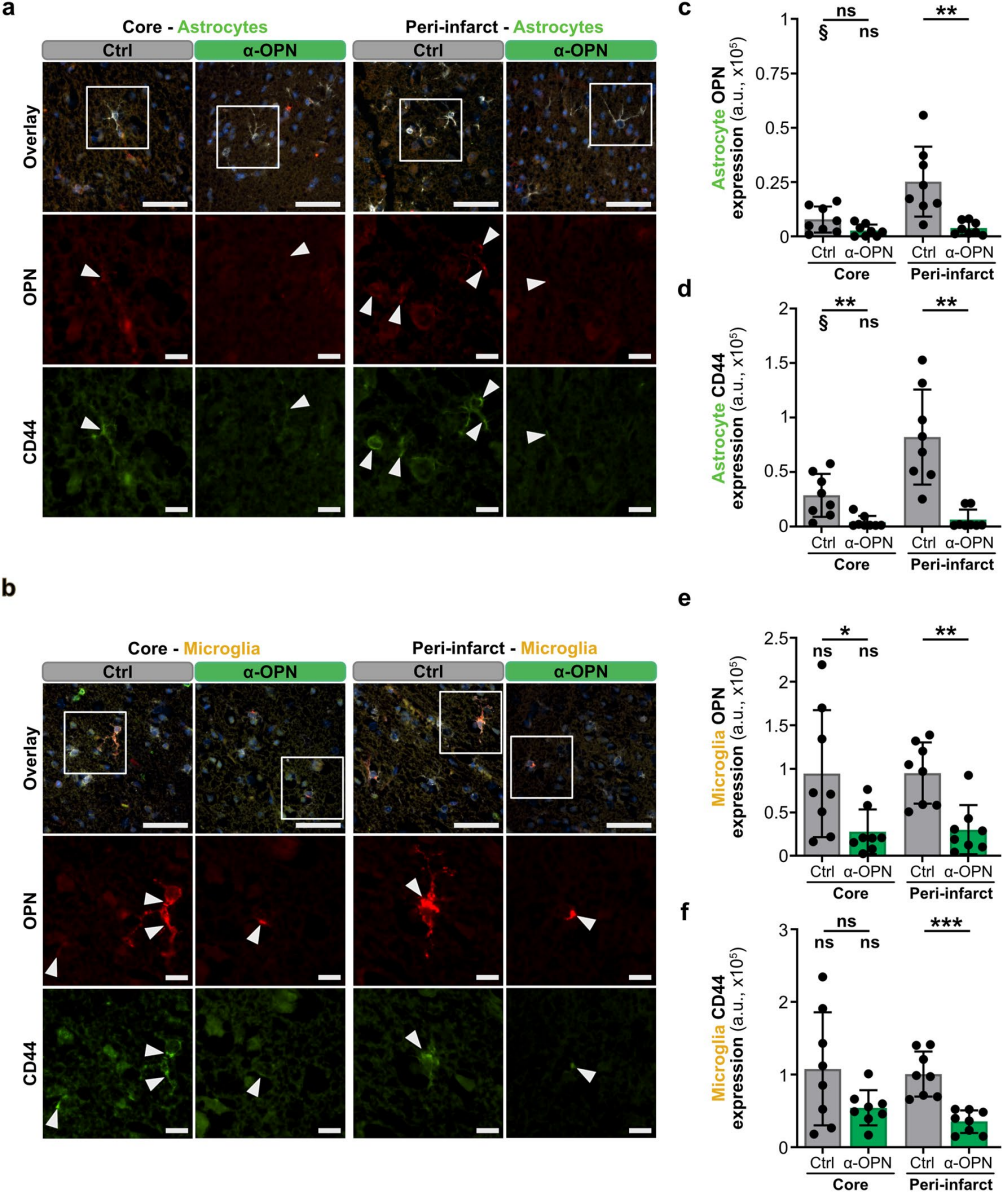
Anti-osteopontin antibody treatment reduces osteopontin and CD44 receptor expression in astrocytes and microglia post-ischemic stroke. (**a**,**b**) Representative images of immunofluorescence staining for osteopontin (OPN, red, inset), CD44 receptor (green, inset) and cell-specific markers (white, overlay) including GFAP for astrocytes (**a**) and IBA1 for microglia/macrophages (**b**) in the infarct core and peri-infarct region of Ctrl IgG and α-OPN antibody-treated mice 24 h post-ischemic stroke. White arrowheads indicate osteopontin and CD44 receptor expression in astrocytes and microglia/macrophages. (**c**–**f**) Quantification of osteopontin and CD44 receptor expression intensity (arbitrary unit, a.u.) in astrocytes (**c**,**d**) and microglia/macrophages (**e**,**f**) in the infarct core and peri-infarct region utilizing 3 images/region/animal, n = 8 and 8 for Ctrl IgG and α-OPN antibody treatment group, respectively; */^§^P < 0.05, **P < 0.01, ***P < 0.001 and not significant (ns) P > 0.05. * indicates two-tailed, unpaired t-test with Welch’s correction when variances were significantly different based on F-test, comparing the two treatment groups for the same region, and § indicates two-tailed, paired t-test comparison of infarct core to peri-infarct region within the same treatment group. Scale bars 50 µm and 10 µm in insets (**a**,**b**).

Our previous work demonstrated that therapeutic neutralization of osteopontin expression in ischemic stroke lesions overall and particularly in peri-infarct NVU cells in the early acute phase of ischemic stroke and clinically significant therapeutic window (4 h post-tMCAO) using a BBB-crossing, anti-osteopontin antibody, improves neurological functions, decreases brain edema volume and reduces the risk for hemorrhagic transformation^12^. Investigation of the time course of osteopontin and CD44 receptor expression, and osteopontin-CD44 interaction in peri-infract and infarct core NVU cells, however, revealed the strongest osteopontin and CD44 receptor expression, and osteopontin-CD44 interaction 15 h post-tMCAO (Fig. 1, Supplementary Fig. S1), prompting us to further optimize the anti-OPN antibody therapy by establishing a combined treatment regimen with anti-osteopontin antibody in the early and late acute phase of ischemic stroke. Therefore, adult WT male mice were subcutaneously injected 4 and 15 h post-stroke either with a polyclonal anti-osteopontin antibody or a control IgG antibody (Fig. 2a). Treatment with anti-osteopontin antibody 4 and 15 h after tMCAO in mice led to improved survival (Fig. 2b), significant improvement in neurological deficit scores, including motor function and reflexes scores (Fig. 2c), and significantly reduced hemorrhagic transformation of stroke lesions (Fig. 2d), edema (Fig. 2e) and infarct volumes (Fig. 2f). Importantly, improved outcome in mice receiving the combined anti-OPN antibody treatment in early and late acute phase was associated with a highly significant reduction of osteopontin and CD44 receptor expression (Figs. 3, 4), and osteopontin-CD44 receptor interaction (Supplementary Figs. S6, S7) in all the individual NVU cell types in the peri-infarct region compared to vehicle-treated animals. This was furthermore associated with significantly decreased expression of glial activation markers GFAP in astrocytes and IBA1 in microglia/macrophages (Supplementary Fig. S7), indicating an inhibitory effect of the anti-OPN antibody therapy on glial activation. In the infarct core, combined anti-OPN antibody treatment in early and late acute phase led to significant reduction of CD44 expression in endothelial cells and astrocytes, osteopontin expression in microglia/macrophages (Figs. 3, 4), and osteopontin-CD44 interaction in astrocytes and microglia/macrophages (Supplementary Fig. S7). However, the therapeutic efficacy of the neutralizing antibody was lower than in the peri-infarct region. Osteopontin and CD44 receptor expression (Supplementary Fig. S8), and consequently their co-localization in NVU cells in the contralateral hemisphere was almost absent and did not differ among groups (Supplementary Fig. S9).

Comparison of efficacy between the combined early and late acute phase therapy versus single early acute phase therapy with anti-osteopontin antibody revealed that combined treatment significantly reduced edema and infarct volumes 24 h post-acute ischemic stroke in mice. Between them, we however did not detect improvement in survival and neurological functions or in risk for hemorrhagic transformation (Supplementary Fig. S10). Reduction of edema and infarct volume in animals with combined administration of anti-osteopontin antibody in early and late acute phases was not associated with a further reduction of osteopontin expression in the individual NVU cell types. Similarly, there was no further decrease of GFAP expression in astrocytes and IBA1 expression in microglia/macrophages in the peri-infarct region and infarct core with the combined targeting in the early acute phases compared to targeting only in the early acute phase. However, in trend, the combined treatment regimen led to lower expression levels of osteopontin particularly in endothelial cells, pericytes and astrocytes, and GFAP in astrocytes in the infarct core at 24 h post-acute ischemic stroke, when compared to the single treatment regimen (Supplementary Fig. S11).

### Therapeutic targeting of osteopontin-CD44 pathway in NVU cells by combined administration of anti-osteopontin antibody in early and late acute phases post-tMCAO ameliorates BBB function in the peri-infarct region and infarct core

**Figure 5:**
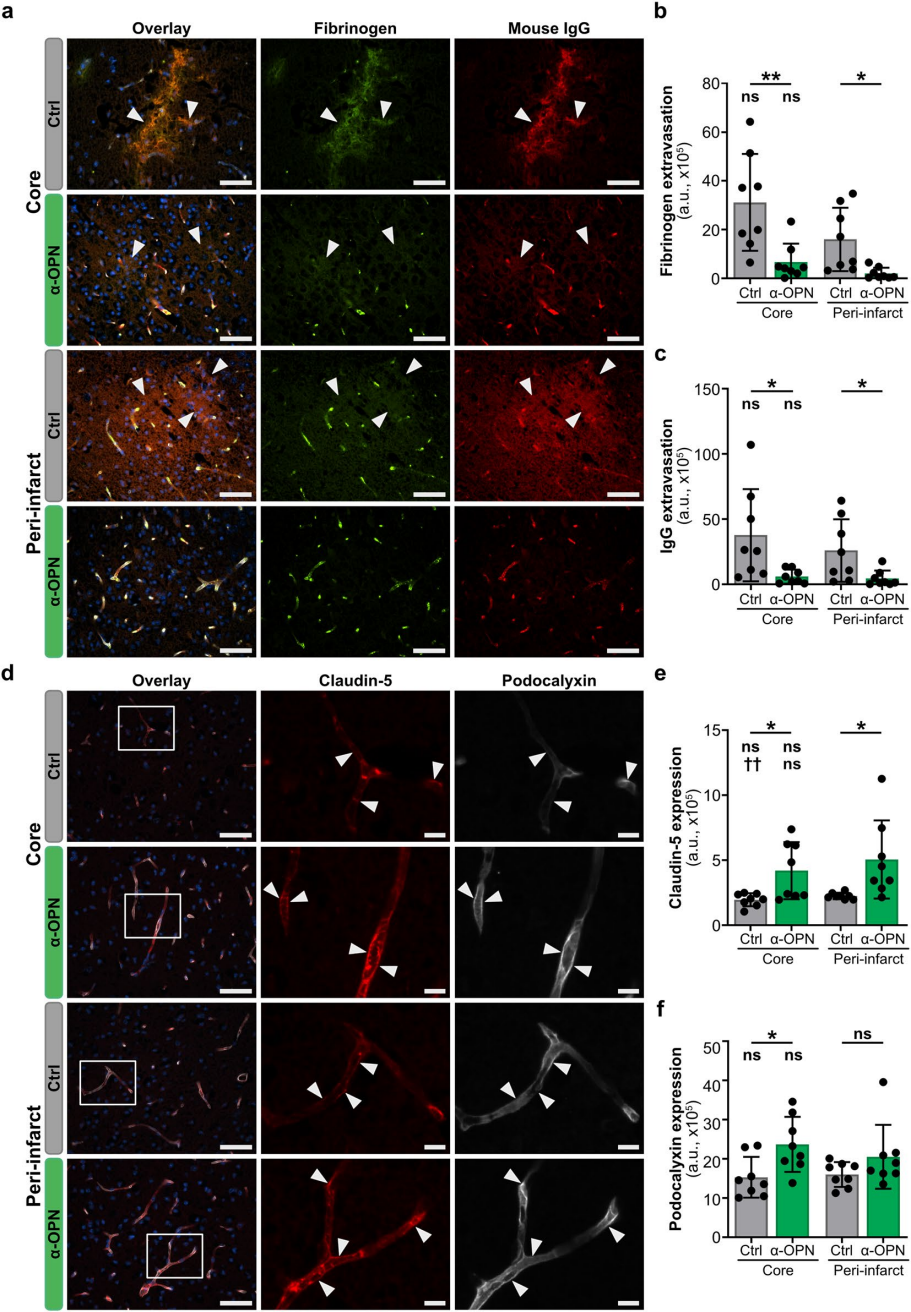
Anti-osteopontin antibody treatment during early and late acute phase of ischemic stroke improves blood–brain barrier function. (**a**–**c**) Representative images of immunofluorescence staining and quantification for fibrinogen (green, **a**,**b**) and mouse immunoglobulin (IgG, red, **a**,**c**) extravasation in the infarct core and peri-infarct region of Ctrl IgG and α-OPN antibody-treated mice 24 h post-ischemic stroke. White arrowheads point to regions of increased fibrinogen and mouse IgG extravasation that are prominent in the infarct core and peri-infarct regions of the control IgG group compared to α-OPN antibody-treated mice. (**d**–**f**) Representative images of endothelial tight junction protein claudin-5 (red, inset) and glycocalyx protein podocalyxin (white, inset) in the infarct core and peri-infarct region of Ctrl IgG and α-OPN antibody-treated mice (**d**) with corresponding quantifications (**e**,**f**). White arrowheads indicate claudin-5 and podocalyxin expression in brain microvascular endothelial cells in the infarct core and peri-infarct region. For quantification three images/region/animal were utilized, n = 8 and 8 for Ctrl IgG and α-OPN antibody treatment group, respectively: *P < 0.05, **P < 0.01 and not significant (ns) P > 0.05. * and ns indicate two-tailed, unpaired t-test with Welch’s correction when variances were significantly different based on F-test, comparing the two treatments groups for the same region, and ns indicates two-tailed, paired t-test comparison of infarct core to peri-infarct region within the same treatment group. Scale bars 50 µm and 10 µm in insets (**a**,**d**).

We previously demonstrated that osteopontin signaling unfolds detrimental effects on BBB in vivo and in vitro and that therapeutic targeting of osteopontin at the NVU 4 h post-tMCAO in mice (single administration of anti-OPN antibody in the early acute phase) improves BBB function in the peri-infarct region, leading to reduced risk for brain edema formation and hemorrhagic transformation in mice with acute ischemic stroke^12^. Since the combined administration of the anti-osteopontin antibody in early and late acute phases more efficiently reduced brain edema volume and additionally reduced stroke tissue damage (Fig. 2e,f, Supplementary Fig. S10), we next sought to evaluate the therapeutic effect of the combined anti-osteopontin antibody treatment on BBB function in ischemic stroke lesions. Compared to controls, reduction of osteopontin in all NVU cells, particularly in endothelial cells, both in the peri-infarct region and infarct core was associated with significantly reduced vascular permeability to fibrinogen and IgG, as indicated by their significantly reduced extravascular signals in these regions (Fig. 5a–c). Decreased permeability was also observed with albumin (Supplementary Fig. S12), a marker for protein leakage assessed commonly by Evans blue dye permeability due to its albumin binding ability^19^. Moreover, increased expression levels of the tight junction protein claudin-5 and the glycocalyx protein podocalyxin (Fig. 5d–f), both of which are required for maintaining BBB function^20,21^, were detected, suggesting that detrimental effects of osteopontin-CD44 pathway on endothelial cells and BBB function are attenuated by the therapeutic antibody administered 4 and 15 h post-tMCAO. In this regard, claudin-5 and podocalyxin expression in endothelial cells of the peri-infarct region and infarct core were similar or even higher compared to those in endothelial cells of the non-affected contralateral hemisphere in the anti-osteopontin antibody treatment group, explaining the low fibrinogen and IgG leakage (Supplementary Fig. S13). Importantly, compared to single administration of anti-osteopontin antibody in the early acute phase, combined administration in early and late acute phases was associated with stronger BBB preserving effects as indicated by reduced extravasation of fibrinogen both in the peri-infarct region and infarct core (Supplementary Fig. S14). IgG leakage was also low both in peri-infarct region and infarct core of animals in both regimens, however it did not differ between them (Supplementary Fig. S14). Both treatment regimens were associated with increased podocalyxin expression in the infarct core and claudin-5 expression in the peri-infarct region, however, combined administration of anti-OPN antibody in early and late acute phases further increased the expression of claudin-5 in the infarct core and podocalyxin in the peri-infarct region (Supplementary Fig. S14).

To prove target engagement and reproduce the inhibitory effect of the neutralizing anti-osteopontin antibody on osteopontin and CD44 receptor expression at gene level we isolated and cultured mouse brain microvascular endothelial cells (MBMEC) for subsequent qRT–PCR experiments. Treatment of MBMEC with either recombinant OPN (0.1 µg/mL) or recombinant osteopontin plus anti-osteopontin antibody revealed that relative *Spp1* and *Cd44* gene expression (Supplementary Fig. S15) were significantly downregulated in the presence of the neutralising antibody used in vivo. These data suggest that osteopontin signaling is indeed affected in brain endothelial cells. Downregulation of *Spp1* and *Cd44*, however, did not affect the expression of *Cdh5* (Supplementary Fig. S15), encoding the endothelial marker VE-Cadherin.

**Figure 6:**
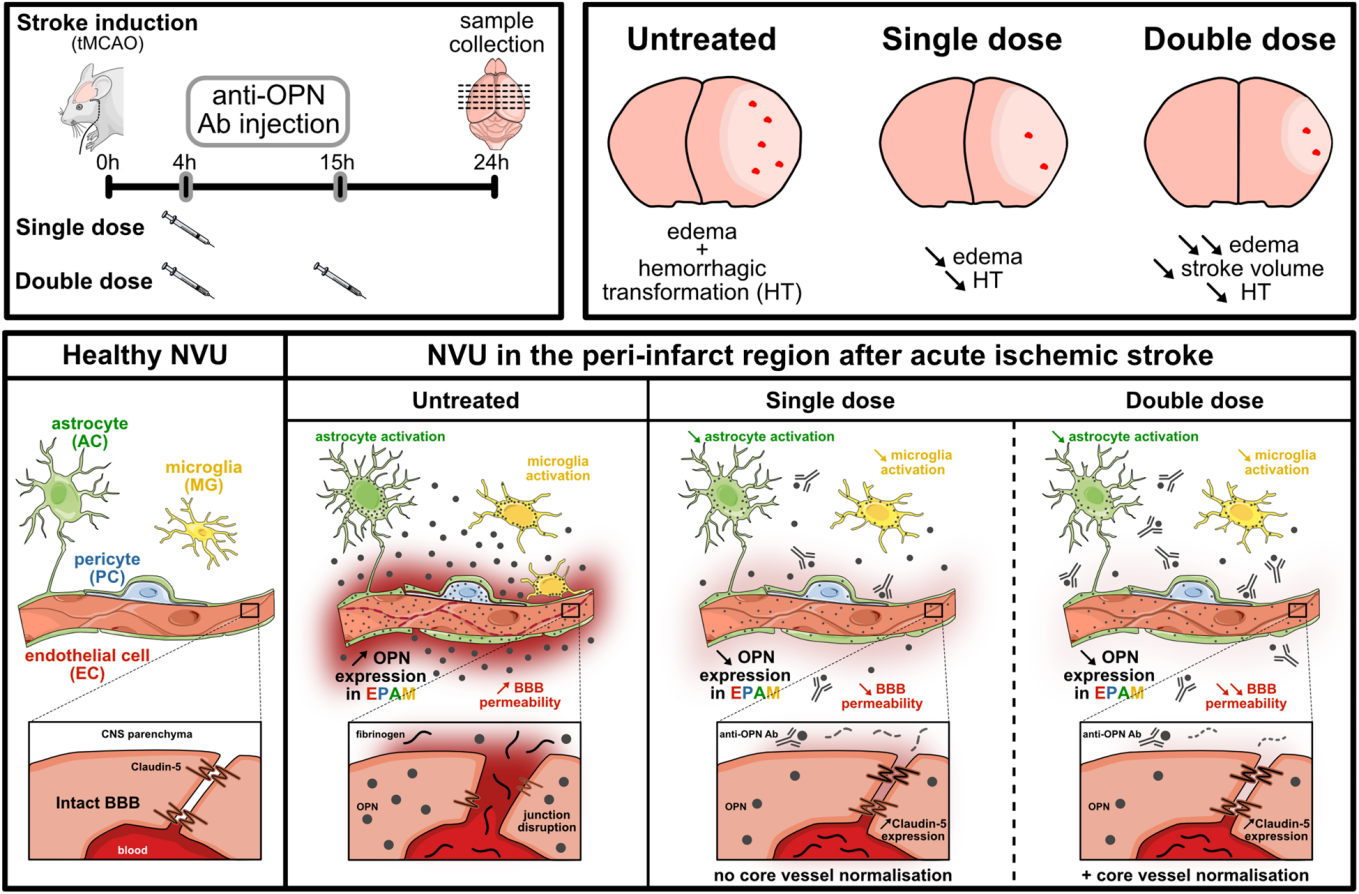
Cerebroprotective effect of antibody-mediated neutralization of dysregulated osteopontin at the neurovascular unit in acute ischemic stroke. Summary schematic depicting the anti-osteopontin antibody treatment regimens (top left panel) 4 h (single treatment duringt early acute phase) or 4 h and 15 h (combined treatment during early and late acute phase) after transient occlusion of middle cerebral artery (tMCAO) and their effects on dysregulated neurovascular unit (NVU) and blood–brain barrier (BBB) function in the ischemic cerebrovascular bed (bottom panel) and on hemorrhagic transformation, cerebral edema formation and stroke volume (top right panel) 24 h post-tMCAO in mice.

Overall, we demonstrate that therapeutic targeting of dysregulated osteopontin at the NVU by administration of a neutralizing anti-osteopontin antibody either during the early acute (single-dose administration, 4 h post-tMCAO) or both in early and late acute phase (combined administration, 4 and 15 h post-tMCAO) is therapeutically effective in a preclinical model of acute ischemic stroke. However, combined osteopontin targeting in the early and late acute phase of ischemic stroke by administration of therapeutic antibody more efficiently improved BBB recovery both in the peri-infarct region and infarct core that is associated with more prominent edema reduction and less stroke tissue damage (Fig. 6).

## Discussion

The treatment of acute ischemic stroke has been revolutionized by the development of reperfusion therapies such as thrombolytic therapy with tPA and mechanical thrombectomy that open occluded cerebral vessels and restore perfusion to the ischemic brain tissue. However, the restoration of blood flow, particularly in the extended therapeutic window, may be associated with cerebral post-ischemic reperfusion injury including severe BBB disruption that contributes to brain edema formation, hemorrhagic transformation, and exacerbation of neuronal tissue damage, all of which can parallel and antagonize the beneficial effects of restoring cerebral circulation after reperfusion therapy^22^. Thus, there is a dire need for effective adjunctive treatments to reperfusion therapy, targeting cerebral injury post-reperfusion^10,23^. In the present study, we demonstrate that therapeutic targeting of osteopontin at the NVU during the early and late acute phase of ischemic stroke improves BBB recovery and reduces both cerebral edema and infarct volume, leading to improved neurological outcome post-acute ischemic stroke.

Ischemic stroke is a major cerebrovascular disorder that is associated with dysfunction of the NVU and the BBB^1^. Targeting of BBB in stroke has been pursued in several preclinical studies, with a majority of them targeting only dysfunctional endothelial cells, the principal cell type forming the BBB^24^. The dysfunction in other cell types of the NVU namely pericytes, astrocytes, and microglia that co-regulate the BBB function along with endothelial cells has not been considered in most of these studies^25^. Along this line, the simultaneous characterization of the dysfunction of several NVU cell types has not been performed either in stroke or in any other neurological disease associated with BBB impairment until recently^12^. In order to characterize NVU dysfunction, we recently established a flow-cytometry based method for simultaneous isolation and analysis of multiple NVU cell-types called EPAM-ia method^12^. As the NVU cell-types together regulate the BBB function, we hypothesized that co-targeting of multiple NVU cell-types in ischemic stroke can lead to robust and effective therapies for this major CNS disease. We tested this hypothesis by obtaining an NVU transcriptomic dataset post stroke applying the EPAM-ia method^12^. Bioinformatic analysis of the dataset using UpSet revealed osteopontin as a novel target commonly dysregulated in multiple NVU cell types, which was also confirmed in human stroke specimen. Therapeutic targeting with an anti-osteopontin antibody improved BBB function that reduced cerebral edema, the risk for hemorrhagic transformation and improved neurological outcome in a mouse model of ischemic stroke. The protective effects of the anti-osteopontin antibody therapy were further confirmed in an OGD model of stroke in vitro using primary mouse brain endothelial cells^12^. In this initial study, we just identified osteopontin and validated it as a therapeutic target for the whole NVU in ischemic stroke. In the current study, we elaborated on the potential of osteopontin targeting in ischemic stroke and further performed downstream mechanistic analysis. In order to obtain an optimized time-dependent regimen for anti-osteopontin therapy, we first evaluated the time course of osteopontin expression post ischemic stroke. These results showed the lowest expression of osteopontin in the early acute phase up to 4 h post stroke and the highest expression in the late acute phase of 15 h post stroke, after which the expression saturated until 24 h, the endpoint of our murine ischemic stroke model of tMCAO (Fig. 1, Supplementary Fig. S1). Similar expression pattern for osteopontin was also observed previously in tubular epithelial cells post renal ischemia reperfusion injury in mice^26^. Along this line, serum osteopontin analysis of stroke patients also show higher levels of osteopontin in acute phase compared to the hyperacute phase^14^. Recently, we demonstrated that a single administration of anti-osteopontin antibody in the therapeutic window of 4 h has a significant therapeutic benefit^12^. We therefore hypothesized that combined administration of anti-osteopontin antibody in the early acute phase (4 h) with that in the late acute phase (15 h) post-stroke, when the osteopontin levels peak might lead to a more significant therapeutic benefit including improvement of infarct volumes along with improvement of edema and neurological outcome.

Indeed, the combined anti-osteopontin IgG therapy in the early and late acute phases led to further improvement in edema compared to single treatment in the early acute phase. Improved BBB function was also indicated by decreased fibrinogen extravasation and increased claudin-5 expression in tight junctions (Supplementary Fig. S14). These data however suggest a rescue of BBB permeability to only large molecules with anti-osteopontin IgG therapy. As increased claudin-5 expression and paracellular permeability were observed in a murine model of temporal lobe epilepsy, the higher claudin-5 levels and improved BBB function post anti-osteopontin IgG therapy in the current study is correlative and needs detailed investigation. In this regard, expression analysis of other junction molecules such as tricellulin, occludin, ZO-1 needs to be performed. Tricellulin is particularly important for its role in controlling permeability of large molecules across the inter-cellular junctions^27^. The assembly of tight junction complexes and its regulation for BBB tightening post anti-osteopontin therapy needs to be demonstrated by studies on BBB ultrastructure. The role of transcellular permeability in ischemic stroke^28^ post anti-osteopontin IgG therapy also needs further investigation.

More importantly, the combined treatment was associated with a reduction in infarct volume that was not observed with the single treatment within the reperfusion window (Fig. 2, Supplementary Fig. S10). As the combined treatment of anti-osteopontin IgG in early and late acute phases further decreased the edema and improved BBB recovery compared to the single treatment in the early acute phase (Supplementary Figs. S10, S14), we suggest that osteopontin neutralization in the acute phase potentially leads to reduced infarct volume due to reduced neuronal damage resulting from BBB impairment. We speculate that there is no direct effect of anti-osteopontin therapy on neurons in the acute phase, which is supported by previous reports showing a beneficial effect of osteopontin in neuronal recovery in the late phases of ischemic stroke^29–31^. Along this line, osteopontin expression was significantly lower both in serum and brain parenchyma in the stroke patients from later phases compared to the ones from the acute phase^12,14^. Improvement of BBB function upon osteopontin neutralization is also supported by our previous study employing anti-osteopontin IgG in OGD settings mimicking stroke in vitro on primary brain endothelial cells^12^. Furthermore, it was previously shown that deficiency of CD44, a receptor for osteopontin is beneficial in ischemic stroke^15^. Along this line, recent in vitro studies provide evidence for improved BBB function upon CD44 inhibition in brain endothelial cells^17^ and increased BBB permeability upon CD44 overexpression in astrocytes co-cultured with brain endothelial cells^18^. Downstream analysis of osteopontin pathway in the current study indicated elevated expression of CD44 in all the NVU cells in the ischemic core and peri-infarct regions compared to the healthy contralateral hemisphere (Fig. 1), which was co-localized with osteopontin suggesting their interaction in BBB function (Supplementary Figs. S6, S7). This is supported by the reduced expression of both osteopontin and CD44 upon treatment with anti-osteopontin IgG both in vitro and in vivo (Figs. 3, 4, Supplementary Fig. S15). Based on our current findings, we suggest osteopontin as a promising therapeutic target in ischemic stroke and specifically suggest anti-ostepontin antibody as a novel adjunctive therapeutic in acute ischemic stroke and potentially other CNS diseases associated with neurovascular unit and blood–brain barrier dysfunction (Fig. 6).

## Methods

### Animals

All in vivo experiments were performed on adult 10-to-12-week-old wild-type (WT) C57BL/6 J male mice (Charles River Laboratories, Sulzfeld, Germany). Three to five animals per cage were housed under standard specific-pathogen-free (SPF) conditions in a temperature-, humidity- and light cycle-controlled facility with free access to food and water. All animals used in this study were sacrificed by cervical dislocation under deep isoflurane anesthesia and their number was kept to a minimum based on extracted tissue/cell amount and statistically appropriate sample size.

Procedures for the use of animals were approved by the local governmental authorities (Regierungspraesidium Darmstadt, Germany) and strictly conducted in accordance to the German Protection of Animals Act and in compliance with the ARRIVE (animal research: reporting of in vivo experiments) guidelines and recommendations in the Guide for Care and Use of Laboratory Animals of the National Institutes of Health.

### Mouse model of transient middle cerebral artery occlusion

Transient middle cerebral artery occlusion (tMCAO) surgeries were performed as described^12,32^. Briefly, mice were analgoanesthetized with 0.1 mg/kg buprenorphine (Temgesic; Essex Pharma, Munich, Germany) and 1.5% isofluorane under spontaneous respiration followed by surgical exposure of the right side extracranial arteries, including the common carotid artery (CCA), external carotid artery (ECA), internal carotid artery (ICA) and pterygopalatine artery (PPA). The ECA and the proximal trunk of the CCA were tied using a 6–0 silk suture and a microclip was placed on the PPA. Next, a small incision into the CCA was carried out using microscissors followed by insertion of a standardized monofilament (Doccol Corporation, #602256PK10) into the ICA. The filament was advanced until resistance was felt where it occludes the proximal middle cerebral artery (MCA). The occluding filament in the CCA was tied to prevent repositioning. The filament was withdrawn after 60 min to allow reperfusion of the ischemic hemisphere. Post-surgery, animals received 0.1 mg/kg buprenorphine every 8 h for pain relief and were allowed to recover on a thermal blanket.

### Therapeutic rescue experiments by administration of anti-osteopontin antibody

Mice were treated at 4 and 15 h after tMCAO by subcutaneous (s.c.) injection of 100 µL PBS containing either polyclonal goat anti-mouse osteopontin antibody (R&D Systems, #AF808) or non-immunized goat immunoglobulin G (R&D Systems, #AB-108-C) at a dose of 0.4 mg/kg body weight, as previously described^12^.

### Assessment of outcome parameters post-acute ischemic stroke

As previously performed analysis did not reveal major changes between tMCAO-operated contralateral and sham-operated ipsilateral brain microvessels^33^, we used contralateral hemispheres from ischemic animals as control instead of sham-operated animals both in the treatment group and control group. Outcome parameters were assessed at 24 h after tMCAO in a blinded fashion. These included survival benefit, global neurological functions, hemorrhagic transformation of ischemic lesions, cerebral edema and infarct volumes.

Survival benefit analysis was performed based on the ratio of surviving and dead animals in both the treatment and control group, and global neurological functions were assessed using the 14-points mNSS^34^ (Supplementary Table S1).

After neurological scoring, all surviving animals were sacrificed and brains were sectioned into three coronal slices using a mouse brain matrix, each of 2 mm thickness. 2,3,5-triphenyltetrazolium chloride (TTC) staining of brain slices was performed to confirm the presence of ischemic stroke by identification of infarcted tissue. Coronal slices of each brain were incubated in 2% TTC (Merck, #108380) in saline at 37 °C for 10 min in the dark and images from both anterior and posterior sides acquired with an optical scanner. TTC positive brain sections were then fixed in 4% paraformaldehyde (PFA) overnight at 4 °C, followed by paraffin embedding using standard protocol and the blocks stored at room temperature to be further used for hematoxylin and eosin (H&E) and immunofluorescence stainings.

Cerebral edema volumes were assessed using TTC stained coronal slices. For both anterior and posterior sides, contralateral and ipsilateral hemispheres were traced and measured using ImageJ software 1.52a. As each cerebrum was sectioned into 2 mm coronal sections, measured areas were multiplied with 1 mm thickness each, obtaining anterior and posterior contralateral and ipsilateral hemisphere volumes. Total contralateral and ipsilateral hemisphere volumes per cerebrum were finally obtained by calculating the sum of volumes across all slices. Cerebral edema volume was then obtained by subtracting the contralateral from the ipsilateral hemisphere volume.

TTC was also used for early confirmation of infarction, however, H&E staining was then used on two sets of 3 µm-thin coronal slices of the same tissues embedded in paraffin with a distance of 1 mm between both sets of coronal slices. H&E stained coronal slices were scanned using a high-resolution scanner (Vectra Polaris, Akoya Biosciences, USA) and exported as TIFF files, which were used for measurement of contralateral and ipsilateral hemispheres and infarct areas (ImageJ software 1.52a). Measured areas were then multiplied with 1 mm thickness each, obtaining contralateral and ipsilateral hemisphere and infarct volumes. Total contralateral and ipsilateral hemisphere and infarct volumes per cerebrum were finally obtained by calculating the sum of volumes across all slices. Edema-adjusted infarct volume [named as stroke volume in the manuscript], was calculated as followed: stroke volume (mm^3^) = infarct volume × (1-[ipsilateral hemisphere volume – contralateral hemisphere volume)/contralateral hemisphere volume])^35^.

Hemorrhagic transformation of ischemic lesions observed in TTC stained slices were characterized as hemorrhagic infarction (petechial infarct) or parenchymal hematoma (hemorrhage with mass effect) using adapted criteria^36^.

Transient intraluminal filament occlusion in C57Bl/6 J mice suffering from dysplasia of posterior communicating arteries leads to an occlusion duration-dependent increase in severity of cerebral hypoperfusion and extension of ischemic stroke beyond MCA territory, such as in the thalamus and the hippocampus^37^. We observed such ischemic strokes beyond MCA territory in 5 mice from the control IgG group and 3 in the anti-OPN antibody treated group. Animals subjected to tMCAO were excluded for any further experiments if one of the following preset exclusion criteria was met: (1) no symptoms of stroke and/or no ischemic lesion visible in triphenyltetrazolium chloride (TTC) staining (1 anti-OPN antibody-treated animal was excluded); (2) intracranial and/or intracerebral hemorrhage due to endoperforation by the monofilament (no animals were excluded); (3) death during anaesthesia and/or surgery but not related to large infarction and/or edema (no animals were excluded).

### Immunofluorescence staining and quantification of paraffin-embedded mouse stroke samples

Immunofluorescence staining and quantification of paraffin-embedded mouse stroke samples was performed as described^12^. 3 µm-thin paraffin-embedded coronal slices of murine stroke tissue samples were deparaffinized and rehydrated in decreasing ethanol concentration according to standard deparaffinization protocol. Heat-induced epitope retrieval using citrate buffer (1 × AR6, PerkinElmer) was performed for 45 min. After cooling down for 30 min at RT, slides were washed with 1 × PBS, followed by blocking/permeabilization in PBS containing 0.5% BSA and 0.2% Triton X-100, pH 7.4 for 1 h. For immunofluorescence staining, tissue sections were incubated in primary antibody solution (0.5% BSA and 0.2% Triton X-100, pH 7.4) overnight at 4 °C. After three washes, 5 min each in 1 × PBS, species-specific fluorescent secondary antibody mix containing DAPI (4′,6-Diamidine-2′-phenylindole dihydrochloride) counterstain (1:500) was added and the slides incubated for 2 h at RT. After three washes in 1 × PBS, slides were mounted with Aqua Polymount (Polysciences Inc.) and allowed to dry at least O/N in dark at RT before proceeding to microscopy. Primary/secondary antibodies and their dilutions are included in Supplementary Table S2. Images were acquired using a wide-field microscope (Nikon 80i) with 40 × objective applying identical exposure and gain settings across samples for a particular antibody combination. For quantification of expression intensity, three coronal sections per sample were used and one image from infarct core, peri-infarct region and contralateral hemisphere per section were acquired as nd2 files (proprietary file format from Nikon Inc. that includes metadata of acquisition) for analysis of immunofluorescence expression intensity. Using the NIS-Elements software (v5, Nikon), raw .nd2 files were subjected to binary thresholding using whole image as the region of interest (ROI). Measurement was then performed for binary area and mean fluorescence intensity. Co-localization analysis was performed from overlapping binary area between corresponding channels using intersection function in the software. All binary layers including intersection layers were stored within the original .nd2 files. Values from each image were exported to a spreadsheet (MS Office Excel). Immunofluorescence expression intensities were obtained in arbitrary units (a.u.) as the product of the binary area and the mean fluorescence intensity of a particular staining within this area. Co-localized expression intensities were obtained as the product of overlapping binary area between channels and the mean intensity of channel of interest. Results were imported to Prism software (v9, GraphPad) for graphing and statistical analysis. For representative images, immunofluorescence microscopy (wide-field—Nikon 80i) was performed using 40 × objective, and images were exported as high-resolution .nd2 files and as TIFF files. Acquisition of images and subsequent expression analyses were performed by a blinded neuropathologist and neurologist.

### Isolation and culture of primary murine brain microvascular endothelial cells

Primary brain microvascular endothelial cells were isolated from murine brains exactly as described previously^12,32^. Murine brains were isolated and rolled on a Whatman filter membrane (Schleicher & Schuell) to peel off meninges. They were subsequently pooled, homogenized in microvessel buffer (15 mM HEPES, 147 mM NaCl, 4 mM KCl, 3 mM CaCl2, 1.2 mM MgCl2, 5 mM glucose, and 0.5% BSA, pH 7.4) using a dounce homogenizer (Wheaton, 0.025 mm clearance) and centrifuged at 400 × g for 10 min at 4 °C. The pellet was then digested with 0.75% collagenase II (Worthington) in buffer A (1:1:1 volume ratio) at 37 °C for 1 h with shaking. For myelin removal, samples were resuspended in 25% BSA and centrifuged at 1500×*g* for 30 min at 4 °C followed by enzymatic digestion of the pellet with Collagenase/Dispase (Roche) and DNase I (Worthington) in buffer A for 15 min at 37 °C. After centrifugation, mouse brain microvascular endothelial cells (MBMEC) were resuspended in MCDB-131 complete medium and seeded on six-well plates precoated with type 1 collagen (150 µg/cm^2^, Corning). After 4 h, the medium was changed to puromycin (5 µg/ml) containing medium, which selects for endothelial cells. The medium was changed back to puromycin-free medium after 3 days.

### Osteopontin and anti-osteopontin IgG treatment of mouse brain endothelial cells

Mouse brain microvascular endothelial cells (MBMEC) at 3 days post isolation were seeded in 24-well plates at passage 1. These cells were treated with recombinant osteopontin (R&D Systems) with or without anti-osteopontin IgG (R&D systems) at the indicated concentrations at 48 h post plating when the cells reached confluency. The treatment period was for 24 h followed by harvesting the cells to obtain RNA and cDNA exactly as described previously^12^ using RNeasy micro kit (Qiagen) followed by cDNA synthesis (Revertaid kit, Thermofisher). qRT-PCR was performed using Absolute qPCR SYBR Green Fluorescein Mix in IQ5 instrument (Biorad) using a previously established PCR protocol^12,32,38^. *Rplp0* was used as a house-keeping gene and qRT-PCR was performed for *Spp1, Cd44* and *Cdh5* using 2^−ΔCt^ method. Primers are listed in Supplementary Table S3.

### Statistics

Data are represented as histogram bars or dot plots with underlying bar graphs showing mean ± SEM (standard error of the mean). The statistical details for each experiment can be found in the figure legend (sample size, testing and P-values). A P-value < 0.05 was considered statistically significant and indicated by the following symbols: */^§^/^†^ for P < 0.05; **/^§§^/^††^ for P < 0.01; ***/^§§§^/^†††^ for P < 0.001 and ****/^§§§§^ for P < 0.0001. Statistical analyses were performed using GraphPad Prism software (v9, GraphPad).

## Supplementary Information

Supplementary Information.

## Data Availability

The data supporting the findings of this study are available from the corresponding author upon reasonable request. Details of the Materials and methods can be found in the Data Supplement S1.
