# Supplementary material for: Anti-osteopontin therapy leads to improved edema and infarct size in a murine model of ischemic stroke
Source: Sci Rep. 2022 Dec 3;12:20925. doi: 10.1038/s41598-022-25245-8 (PMC9719559; doi:10.1038/s41598-022-25245-8)
Supplement: Supplementary file 1 — Supplementary Information. [file 41598_2022_25245_MOESM1_ESM.pdf]

Anti-osteopontin therapy leads to improved edema and infarct size in a murine model of ischemic stroke

Daniel Spitzer,[1, 2, 8] Tim Puetz,[1, 2] Moritz Armbrust,[1] Maika Dunst,[1] Jadranka Macas,[1, 3, 5] Florian Croll, [1] Karl-Heinz Plate,[1, 3, 4, 5, 6, 8] Yvonne Reiss,[1, 3, 4, 5, 8] Stefan Liebner,[1, 6, 7, 8] Patrick N. Harter,[1, 3, 4, 5, 8] Sylvaine Guérit,[1] and Kavi Devraj[1, 5, 8 *]

Author affiliations:

1 Edinger Institute (Institute of Neurology), Goethe University Hospital, Frankfurt, 60528, Germany

2 Department of Neurology, Goethe University Hospital, Frankfurt, 60528, Germany

3 German Cancer Consortium (DKTK) Partner site Frankfurt/Mainz, 60528, Frankfurt,

4 Germany and German Cancer Research Center (DKFZ), Heidelberg, 69120, Germany

5 Frankfurt Cancer Institute (FCI), Goethe University Hospital, Frankfurt, 60528, Germany

6 German Center for Cardiovascular Research (DZHK), Partner Site Frankfurt/Mainz, 60528, Germany

7 Excellence Cluster Cardio Pulmonary System (CPI), Partner Site Frankfurt, Frankfurt, 60528, Germany

8 Center for Personalized Translational Epilepsy Research (CePTER), Frankfurt, 60528, Germany

*Correspondence: Dr. Kavi Devraj; E-mail: kdevraj@uni-frankfurt.de

Supplemental Material

Supplemental Methods

Animals

Adult (10-12 weeks old) wild-type C57BL/6J male mice (Charles River Laboratories, Sulzfeld, Germany) used in this study were housed in groups of three to five per cage under standard specific-pathogen-free (SPF) conditions in a temperature-, humidity- and light cycle-controlled facility (20 ± 2°C; 50 ± 10%; 12 hours light/dark cycle) with free access to food and water. All animals were sacrificed by cervical dislocation under deep isoflurane anesthesia and their number was kept to a minimum based on extracted tissue/cell amount and statistically appropriate sample size. All experiments using animals were strictly conducted in accordance to the German Protection of Animals Act and in compliance with the ARRIVE (animal research: reporting of in vivo experiments) guidelines and recommendations in the Guide for Care and Use of Laboratory Animals of the National Institutes of Health, and were approved by the local governmental authorities (Regierungspraesidium Darmstadt, Germany; approval number FK/1052).

Supplemental Figures

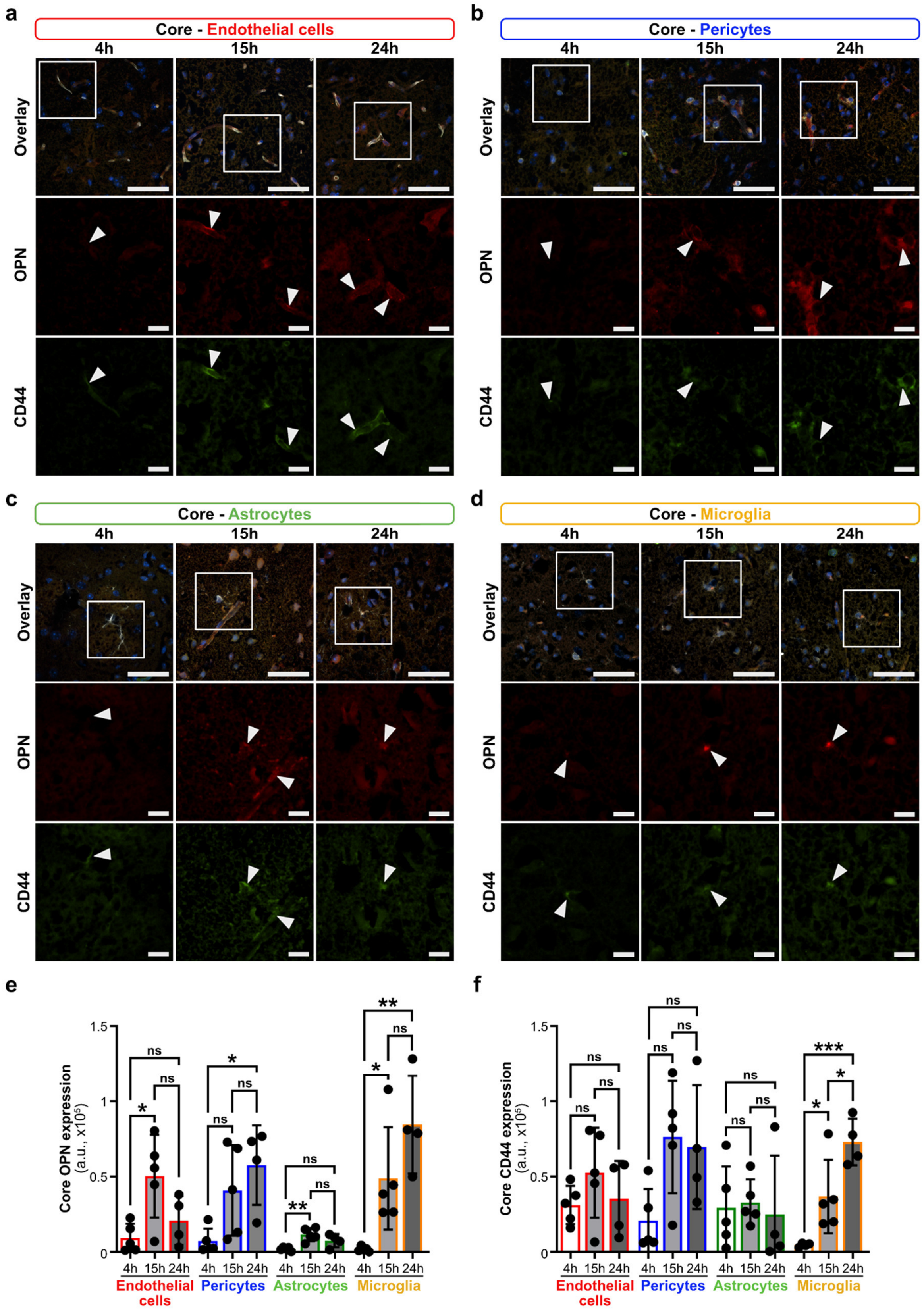

Supplementary Figure 1. Time course of osteopontin and CD44 receptor expression in murine NVU cells in the infarct core post-acute ischemic stroke. a-d, Representative images of immunofluorescence staining for osteopontin (OPN, red, inset), CD44 receptor (green, inset) and cell-specific markers (white, overlay) including podocalyxin for endothelial cells (**a**), CD13 for pericytes (**b**), GFAP for astrocytes (**c**) and IBA1 for microglia/macrophages (**d**) in the infarct core of mice 4 h (early acute phase), and 15 hours and 24 hours (late acute phase) post-tMCAO. White arrowheads indicate OPN and CD44 receptor expression in NVU cells. **e** and **f,** Quantification of OPN and CD44 receptor expression intensity (arbitrary unit, a.u.) in NVU cells in the infarct core at indicated time points utilizing 3 images/region/animal, n=5 (4h), n=5 (15 hours) and n=4 (24 hours); *P<0.05, **P<0.01, ***P<0.001, and not significant (ns) P>0.05 by one-way ANOVA with Tukey's multiple comparisons test. Scale bars: 50 μm in overlays and 10 μm in insets (**a-d**).

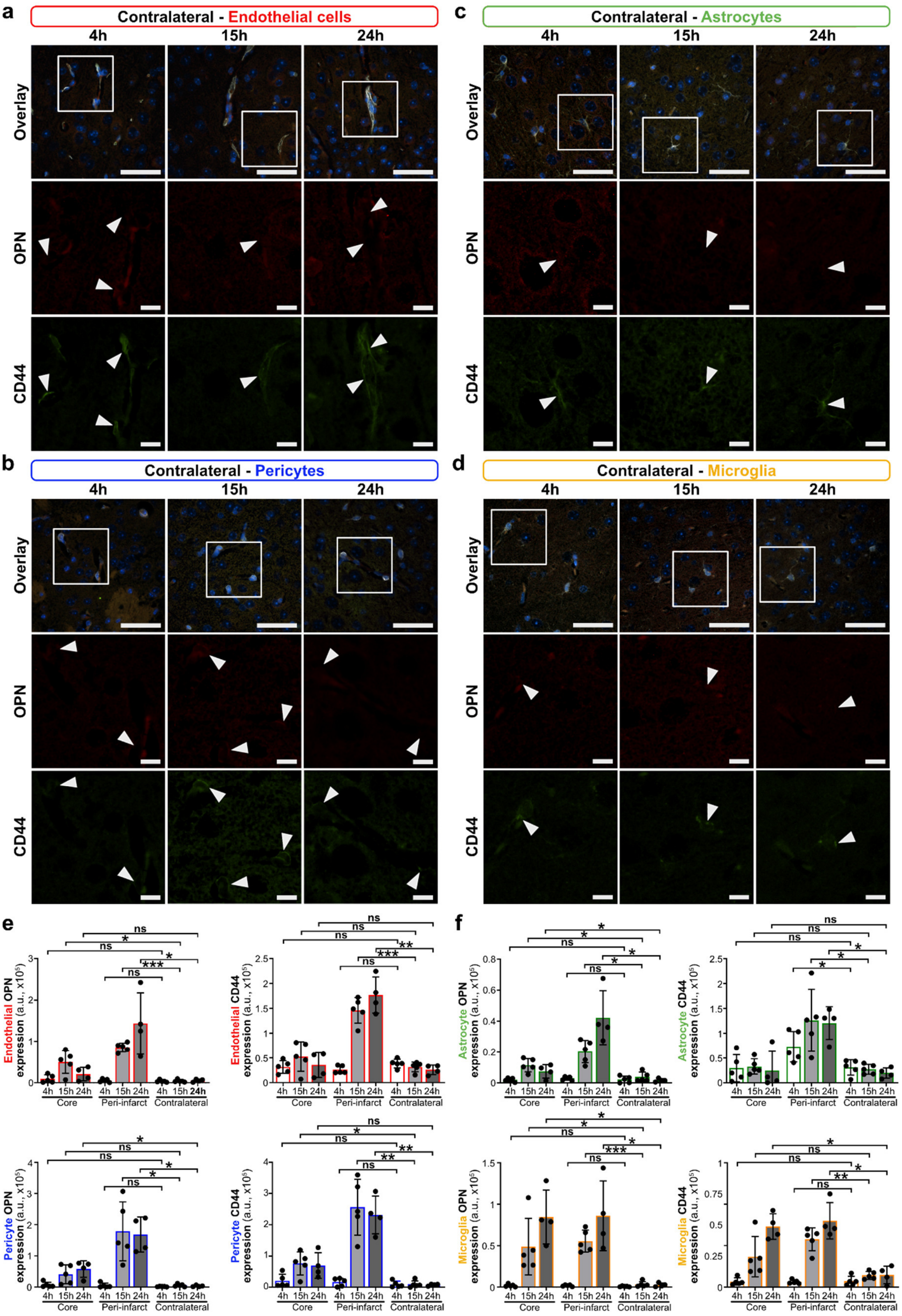

a
Contralateral - Endothelial cells
b
Contralateral - Pericytes
c
Contralateral - Astrocytes
d
Contralateral - Microglia
4h
15h
24h
Overlay
OPN
CD44
e
f
Core
Peri-infarct
Contralateral
Endothelial OPN expression (a.u., x10⁵)
Endothelial CD44 expression (a.u., x10⁵)
Pericyte OPN expression (a.u., x10⁵)
Pericyte CD44 expression (a.u., x10⁵)
Astrocyte OPN expression (a.u., x10⁵)
Astrocyte CD44 expression (a.u., x10⁵)
Microglia OPN expression (a.u., x10⁵)
Microglia CD44 expression (a.u., x10⁵)

Supplementary Figure 2. Time course of osteopontin and CD44 receptor expression in NVU cells in contralateral hemisphere, infarct core and peri-infarct region. a-d, Representative images of immunofluorescence staining for osteopontin (OPN, red, inset), CD44 receptor (green, inset) and cell-specific markers (white, overlay) including podocalyxin for endothelial cells (**a**), CD13 for pericytes (**b**), GFAP for astrocytes (**c**) and IBA1 for microglia/macrophages (**d**) in the contralateral hemisphere 4 hours (early late phase), and 15 hours and 24 hours (late acute phase) after ischemic stroke. White arrowheads indicate OPN and CD44 receptor expression in neurovascular unit cells. **e** and **f,** Quantification of OPN and CD44 receptor expression intensity (arbitrary unit, a.u.) in core, peri-infarct and contralateral endothelial cells (**e**, top panel), pericytes (**e**, bottom panel), astrocytes (**f**, top panel) and microglia/macrophages (**f**, bottom panel) at indicated time points utilizing 3 images/region/animal, n=5 (4 hours), n=5 (15 hours) and n=4 (24 hours); *$P<0.05$, **$P<0.01$, ***$P<0.001$ and not significant (ns) $P>0.05$ by two-tailed, paired t-test, comparing infarct core and peri-infarct region with contralateral hemisphere 4 hours, 15 hours and 24 hours post-ischemic stroke, respectively. Scale bars: 50 μm in overlays and 10 μm in insets (**a-d**).

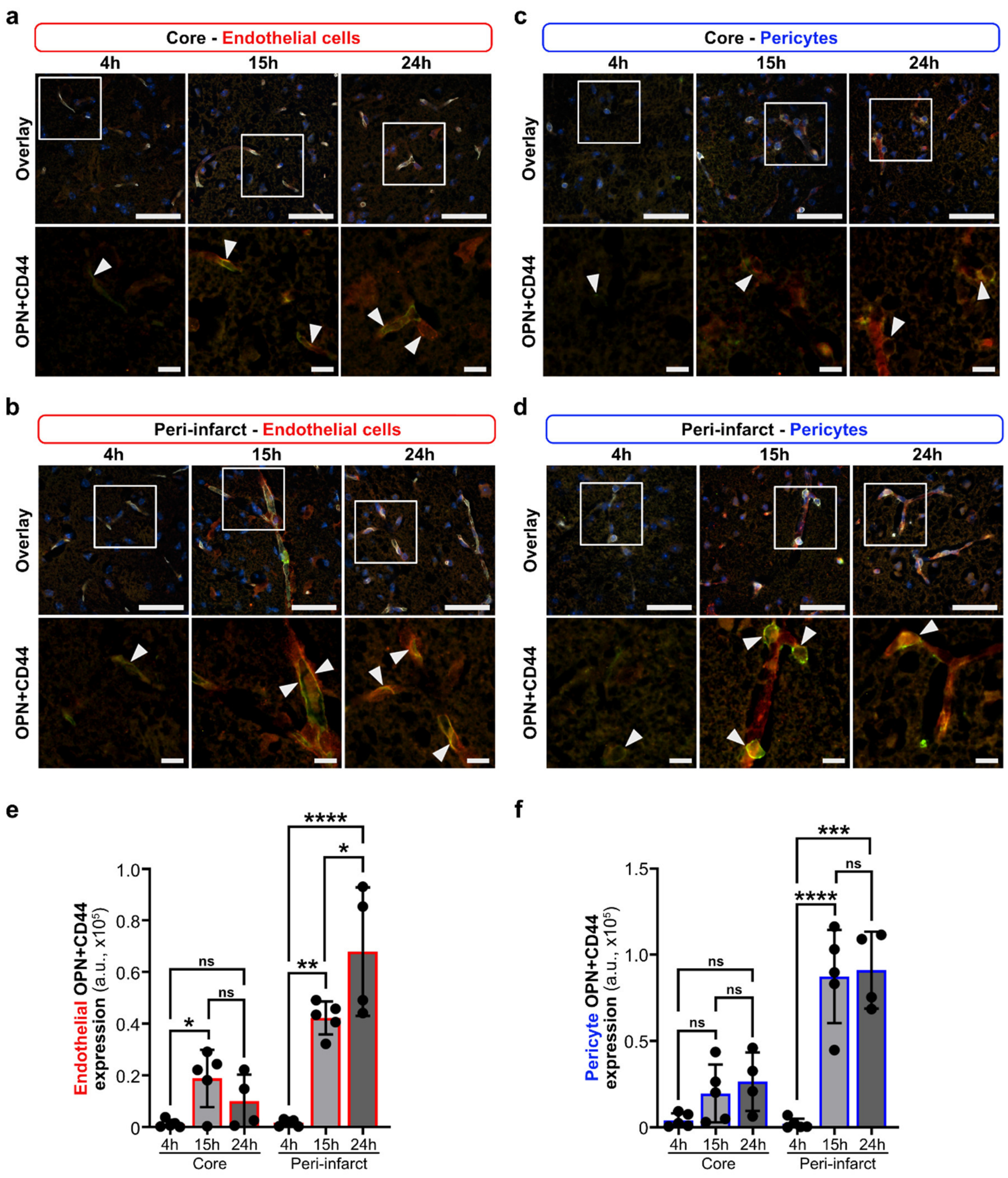

Supplementary Figure 3. Time course of co-localized osteopontin and CD44 receptor expression in microvascular endothelial cells and pericytes after acute ischemic stroke. a-d, Representative images of immunofluorescence staining for co-localized OPN and CD44 receptor (OPN+CD44, yellow, inset) and cell-specific markers (white, overlay) including podocalyxin for endothelial cells (**a** and **b**) and CD13 for pericytes (**c** and **d**), suggesting interaction between OPN and its cell surface receptor CD44 in endothelial cells and pericytes in the infarct core and peri-infarct region of mice 4 hours (early acute phase), and 15 hours and 24 hours (late acute phase) post-stroke. White arrowheads indicate co-localized OPN and CD44 receptor in endothelial cells and pericytes. **e** and **f**, Quantification of co-localized OPN and

CD44 receptor expression intensity (arbitrary unit, a.u.) in endothelial cells (**e**) and pericytes (**f**) in the ischemic core and peri-infarct region at indicated time points utilizing 3 images/region/animal, n=5 (4 hours), n=5 (15 hours) and n=4 (24 hours); *P<0.05, **P<0.01, ***P<0.001, ****P<0.0001 and not significant (ns) P>0.05 by one-way ANOVA with Tukey's multiple comparisons test. Scale bars: 50 µm in overlays and 10 µm in insets (**a-d**).

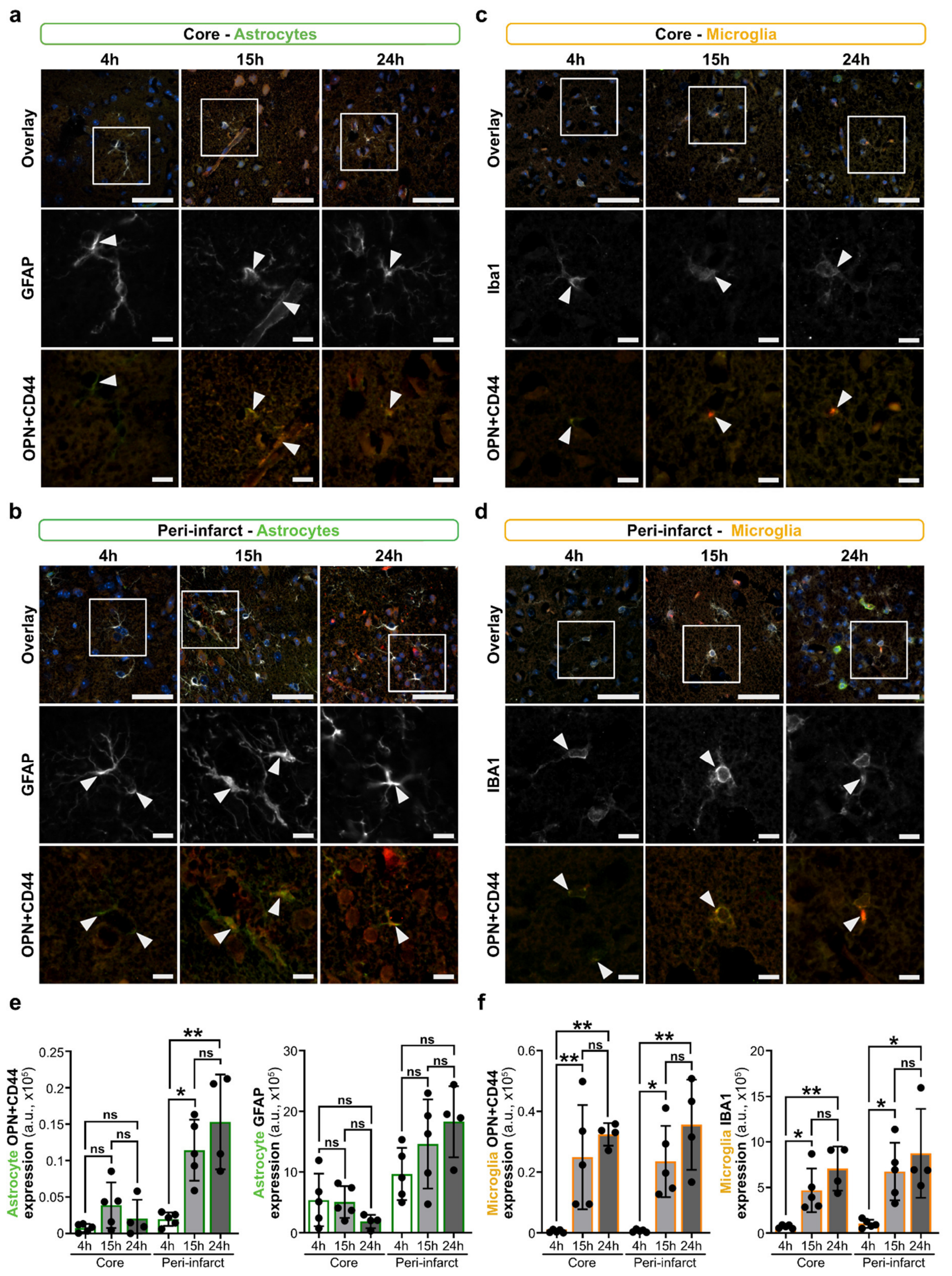

Supplementary Figure 4. Time course of co-localized osteopontin and CD44 receptor expression in astrocytes and microglia/macrophages after acute ischemic stroke. a-d, Representative images of immunofluorescence staining for co-localized osteopontin and CD44 receptor (OPN+CD44, yellow, inset) and cell-specific markers (white, overlay and inset)

including GFAP for astrocytes (**a** and **b**) and IBA1 for microglia/macrophages (**c** and **d**), suggesting interaction between OPN and cell surface receptor CD44 in astrocytes and microglia/macrophages in the infarct core and peri-infarct region of mice 4 hours (early acute phase), and 15 hours and 24 hours (late acute phase) post-stroke. White arrowheads indicate co-localized OPN and CD44 receptor in astrocytes and microglia/macrophages. **e** and **f,** Quantification of co-localized OPN and CD44 receptor and cell-specific marker expression intensity (arbitrary unit, a.u.) including GFAP for astrocytes (**e**) and IBA1 for microglia/macrophages (**f**) in ischemic core and peri-infarct region at indicated time points utilizing 3 images/region/animal, n=5 (4 hours), n=5 (15 hours) and n=4 (24 hours); *$P<0.05$, **$P<0.01$ and not significant (ns) $P>0.05$ by one-way ANOVA with Tukey's multiple comparisons test. Scale bars: 50 μm in overlays and 10 μm in insets (**a-d**).

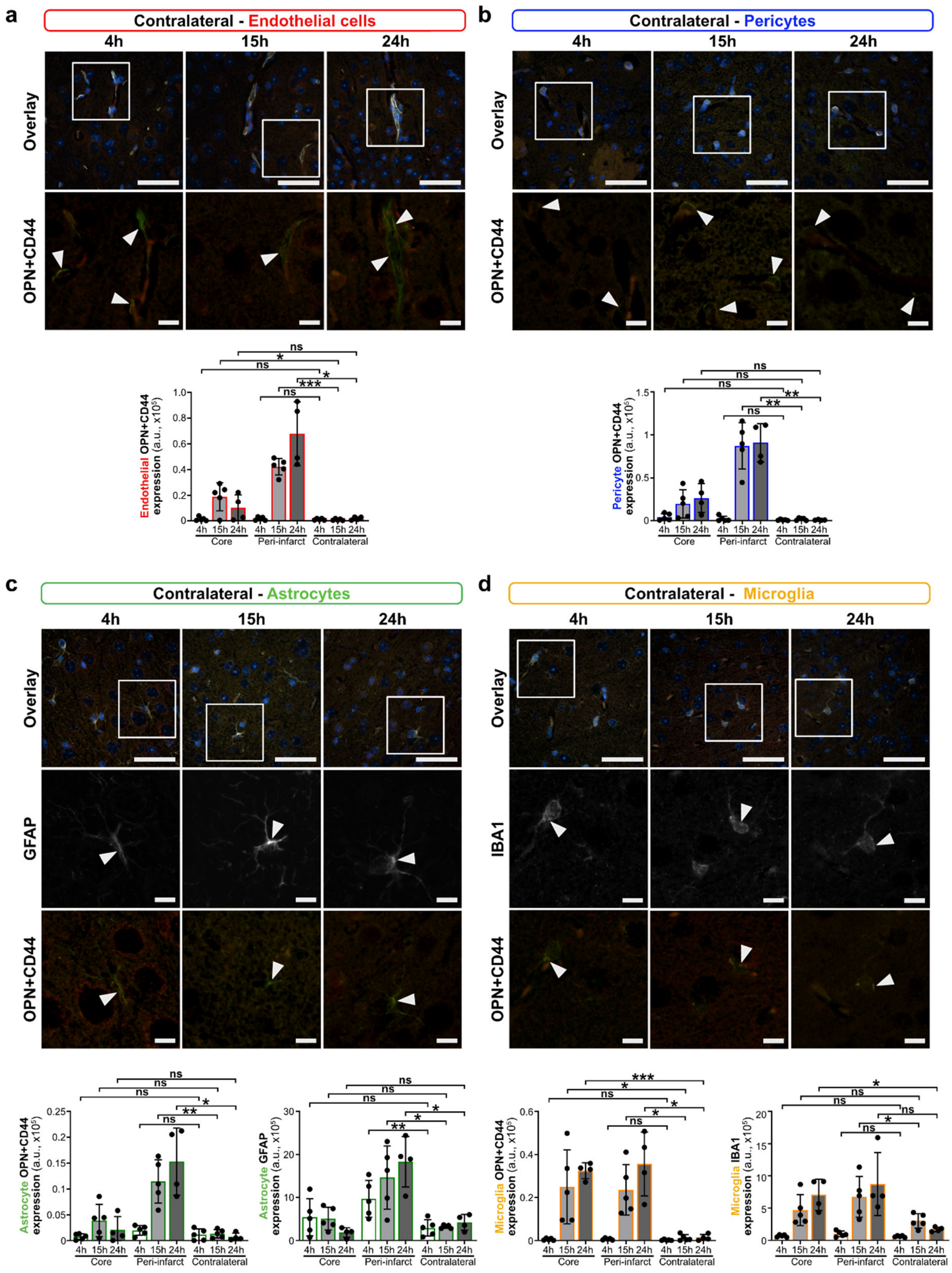

Supplementary Figure 5. Time course of co-localized osteopontin and CD44 receptor expression in neurovascular unit cells in contralateral hemisphere, infarct core and peri-infarct region. Representative images of immunofluorescence staining for co-localized osteopontin and CD44 receptor (OPN+CD44, yellow, inset) and cell-specific markers (white,

overlay in A-D, and inset in C and D) including podocalyxin for endothelial cells (**a**, top panel), CD13 for pericytes (**b**, top panel), GFAP for astrocytes (**c**, top panel) and IBA1 for microglia/macrophages (**d**, top panel), suggesting interaction between OPN and cell surface receptor CD44 in contralateral hemisphere 4 hours (early acute phase), and 15 hours and 24 hours (late acute phase) after ischemic stroke. White arrowheads indicate co-localized OPN and CD44 receptor in neurovascular unit cells. Quantification of co-localized OPN and CD44 receptor expression intensity (arbitrary unit, a.u.) in infarct core, peri-infarct and contralateral endothelial cells (**a**, bottom panel), pericytes (**b**, bottom panel), astrocytes (**c**, bottom left panel) and microglia/macrophages (**d**, bottom left panel) at indicated time points. Quantification of GFAP in astrocytes (**c**, bottom right panel) and IBA1 in microglia/macrophages (**d**, bottom right panel) in the infarct core, peri-infarct region and contralateral hemisphere at indicated time points. For quantification three images/region/animal were utilized, n=5 (4 hours), n=5 (15 hours) and n=4 (24 hours); *$P<0.05$, **$P<0.01$, ***$P<0.001$ and not significant (ns) $P>0.05$ by two-tailed, paired t-test, comparing infarct core or peri-infarct region with contralateral hemisphere 4 hours, 15 hours and 24 hours post-ischemic stroke, respectively. Scale bars: 50 μm in overlays and 10 μm in insets (**a-d**).

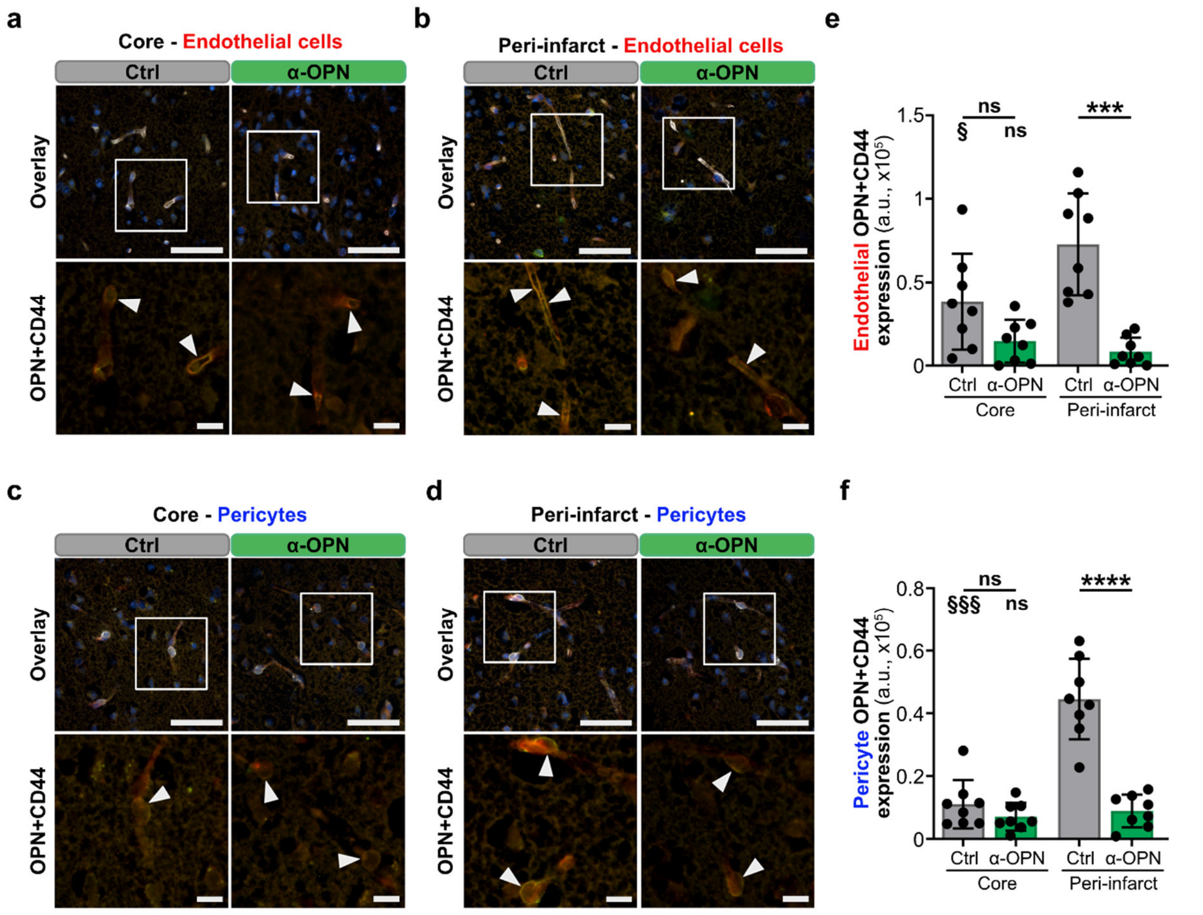

Supplementary Figure 6. Anti-osteopontin antibody treatment reduces co-localized osteopontin and CD44 receptor expression in endothelial cells and pericytes after ischemic stroke. a-d, Representative images of immunofluorescence staining for co-localized osteopontin and CD44 (OPN+CD44, yellow, inset) and cell-specific markers (white, overlay) including podocalyxin for endothelial cells (**a** and **b**) and CD13 for pericytes (**c** and **d**), suggesting interaction between OPN and its cell surface receptor CD44 in endothelial cells and pericytes in the infarct core and peri-infarct region of Ctrl IgG and α-OPN antibody-treated mice 24 hours post-ischemic stroke. White arrowheads indicate co-localized OPN and CD44 receptor in endothelial cells and pericytes. **e** and **f**, Quantification of co-localized OPN and CD44 receptor expression intensity (arbitrary unit, a.u.) in core and peri-infarct endothelial cells (**e**) and pericytes (**f**) utilizing 3 images/region/animal, n=8 and 8 for Ctrl IgG and α-OPN antibody treatment group, respectively; §$P<0.05$, ***/§§§$P<0.001$, ****$P<0.0001$ and not significant (ns) $P>0.05$. * indicates two-tailed, unpaired t-test with Welch's correction when variances were significantly different based on F-test, comparing the two treatments groups for the same region, and § indicates two-tailed, paired t-test comparison of infarct core to peri-

infarct region within the same treatment group. Scale bars: 50 μm in overlays and 10 μm in insets (**a-d**).

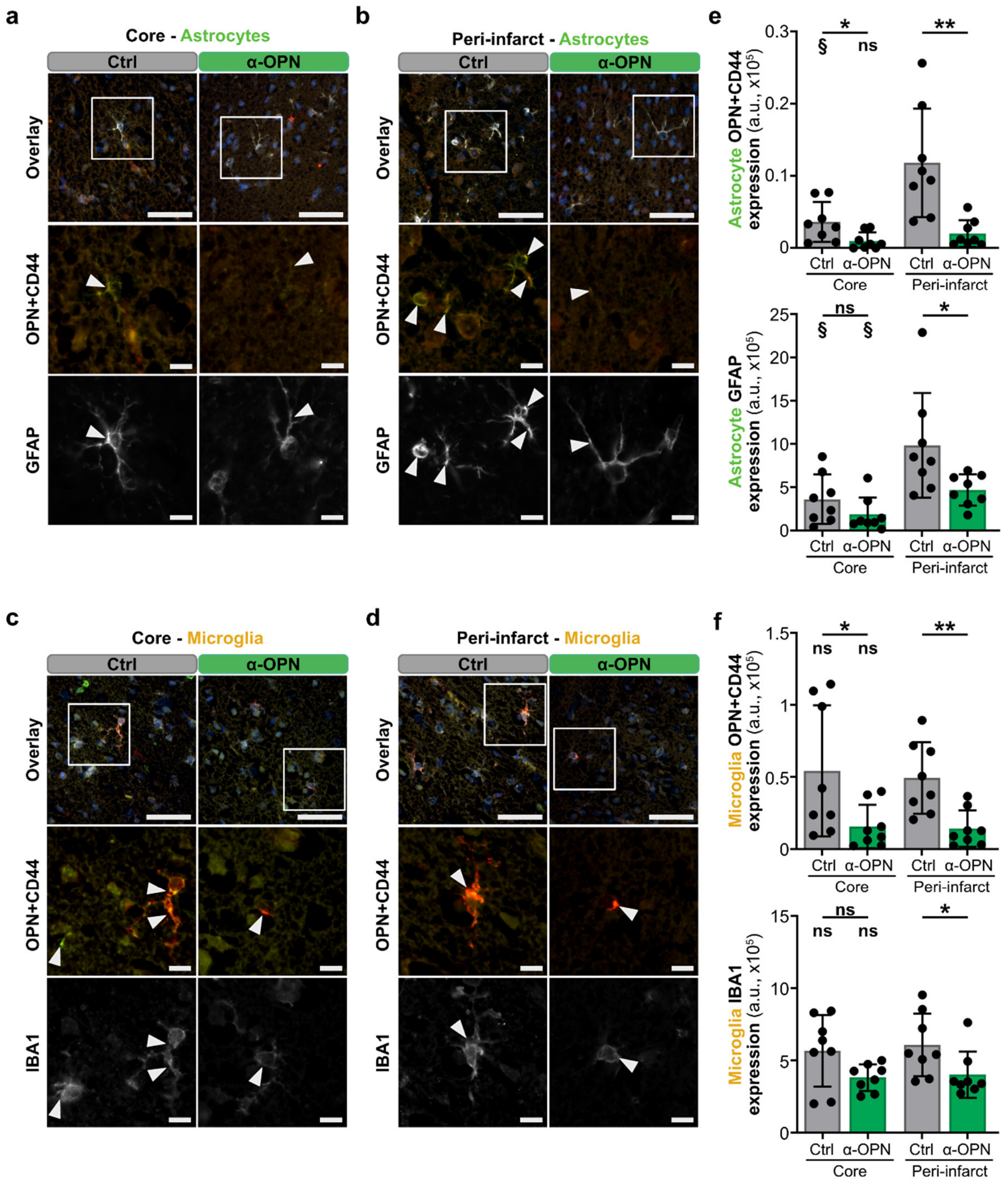

Supplementary Figure 7. Anti-osteopontin antibody treatment reduces co-localized osteopontin and CD44 receptor expression in astrocytes and microglia/macrophages post-acute ischemic stroke. a-d, Representative images of immunofluorescence staining for co-localized osteopontin and CD44 (OPN+CD44, yellow, inset) and cell-specific markers (white, overlay and inset) including GFAP for astrocytes (**a** and **b**) and IBA1 for microglia/macrophages (**c** and **d**), suggesting interaction between OPN and cell surface receptor CD44 in astrocytes and microglia/macrophages in the infarct core and peri-infarct region of Ctrl IgG and α-OPN antibody-treated mice 24 hours post-stroke. White arrowheads indicate co-localized OPN and CD44 receptor in astrocytes and microglia/macrophages. **e** and **f**,

Quantification of co-localized OPN and CD44 receptor, and cell-specific marker expression intensity (arbitrary unit, a.u.) including GFAP for astrocytes (**e**) and IBA1 for microglia/macrophages (**f**) in the infarct core and peri-infarct region utilizing 3 images/region/animal, n=8 and 8 for Ctrl IgG and α-OPN antibody treatment group, respectively; */§$P<0.05$, **$P<0.01$ and not significant (ns) $P>0.05$. * indicates two-tailed, unpaired t-test with Welch's correction when variances were significantly different based on F-test, comparing the two treatments groups for the same region, and § indicates two-tailed, paired t-test comparison of infarct core to peri-infarct region within the same treatment group. Scale bars: 50 μm in overlays and 10 μm in insets (**a-d**).

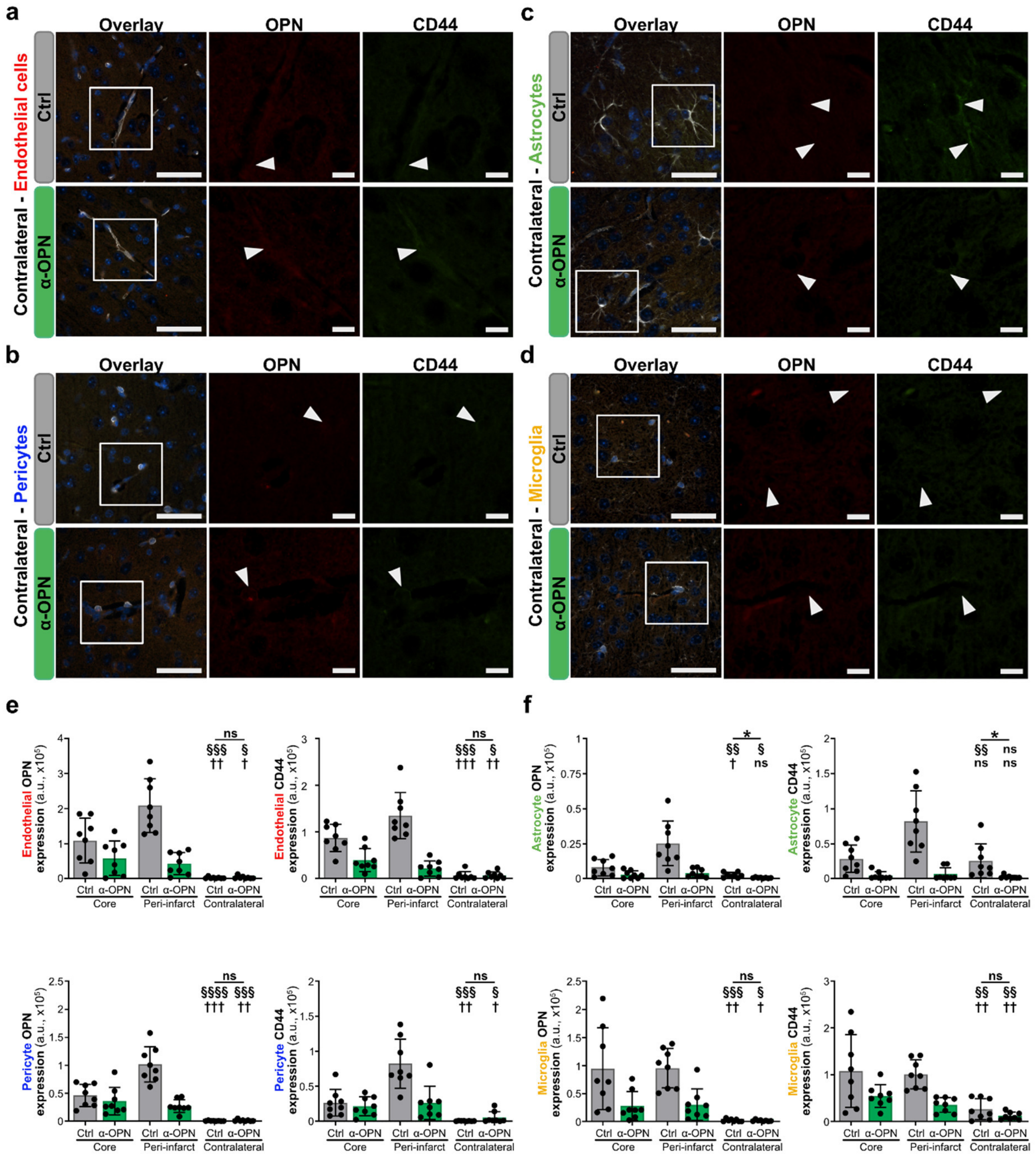

Supplementary Figure 8. Osteopontin and CD44 receptor expression in neurovascular unit cells in contralateral hemisphere, infarct core and peri-infarct region following anti-osteopontin antibody treatment. a-d, Representative images of immunofluorescence staining for osteopontin (OPN, red, inset), CD44 (green, inset) and cell-specific markers (white, overlay) including podocalyxin for endothelial cells (**a**), CD13 for pericytes (**b**), GFAP for astrocytes (**c**) and IBA1 for microglia/macrophages (**d**) in the contralateral hemisphere of Ctrl IgG and α-OPN antibody-treated mice 24 hours post-ischemic stroke. White arrowheads indicate OPN and CD44 expression in neurovascular unit cells. **e** and **f**, Quantification of OPN and CD44 receptor expression intensity (arbitrary unit, a.u.) in core, peri-infarct and contralateral endothelial cells

(**e**, top panel), pericytes (**e**, bottom panel), astrocytes (**f**, top panel) and microglia/macrophages (**f**, bottom panel) utilizing 3 images/region/animal, n=8 and 8 for Ctrl IgG and α-OPN antibody treatment group, respectively; */§/†P<0.05, §§/††P<0.01, §§§/†††P<0.001, §§§§P<0.0001 and not significant (ns) P>0.05. * indicates two-tailed, unpaired t-test, with Welch's correction when variances were significantly different based on F-test, comparing the two treatment groups for the same region, § indicates two-tailed, paired t-test comparison of peri-infarct to contralateral regions within the same treatment group/animal, and † indicates two-tailed, paired t-test comparison of core to contralateral regions within the same treatment group/animal. Scale bars: 50 μm in overlays and 10 μm in insets (**a-d**).

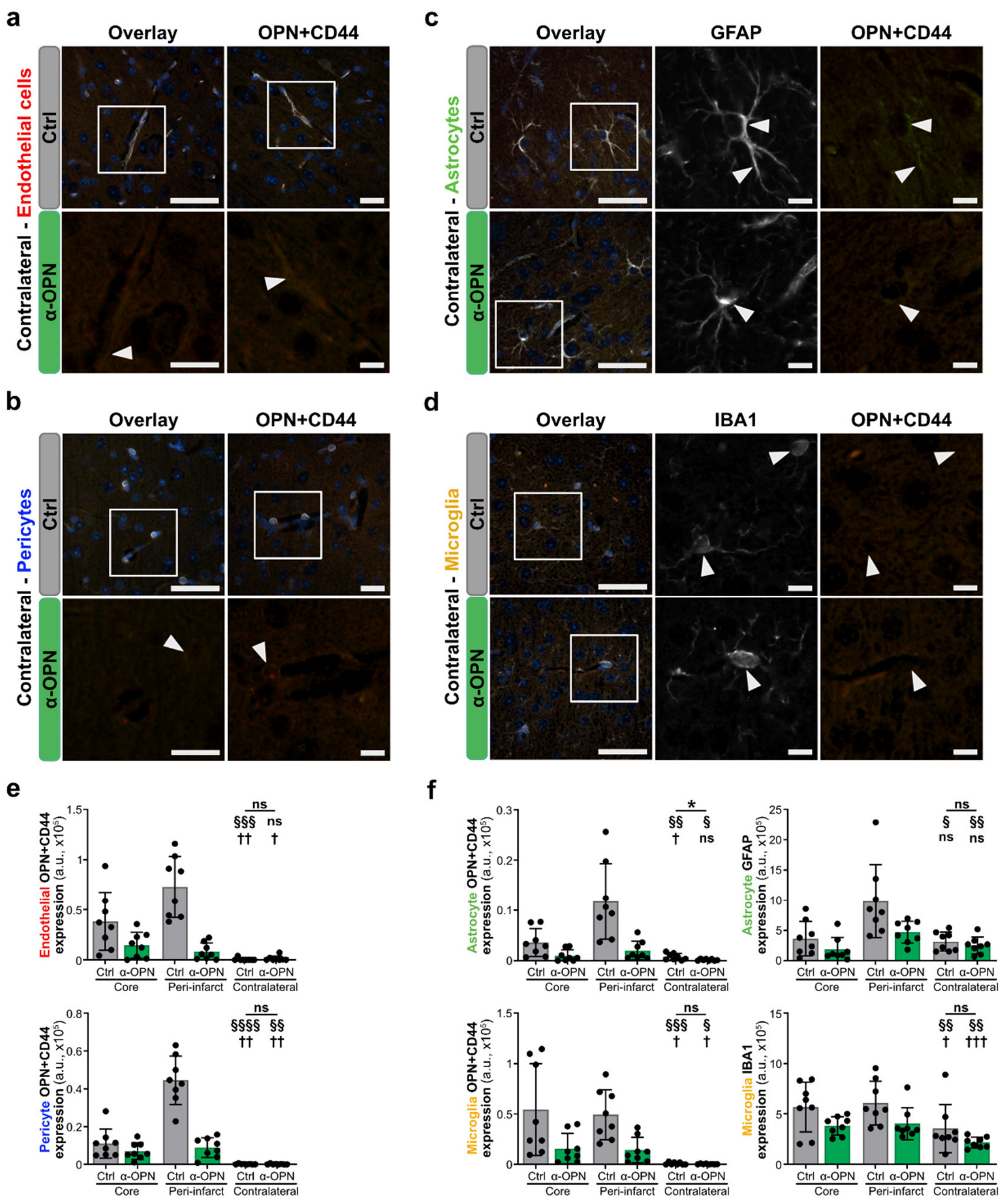

Supplementary Figure 9. Osteopontin and CD44 receptor expression in neurovascular unit cells in contralateral hemisphere, infarct core and peri-infarct region after administration of anti-osteopontin antibody. a-d, Representative images of immunofluorescence staining for co-localized osteopontin and CD44 (OPN+CD44, yellow, inset) and cell-specific markers (white, overlay in A-D, and inset in C and D) including podocalyxin for endothelial cells (**a**), CD13 for pericytes (**b**), GFAP for astrocytes (**c**) and IBA1 for microglia/macrophages (**d**) in the contralateral hemisphere of Ctrl IgG and α-OPN antibody-treated mice 24 hours post-stroke. White arrowheads indicate co-localized OPN and CD44 receptor in neurovascular unit cells. **e** and **f**, Quantification of co-localized OPN and CD44 receptor expression intensity (arbitrary unit, a.u.) in infarct core, peri-infarct and contralateral

endothelial cells (**e**, top panel), pericytes (**e**, bottom panel), astrocytes (**f**, top left panel) and microglia/macrophages (**f**, bottom left panel), and quantification (arbitrary unit, a.u.) of GFAP in astrocytes (**e**, top right panel) and IBA1 in microglia/macrophages (**f**, bottom right panel) in infarct core, peri-infarct region and contralateral hemisphere utilizing 3 images/region/animal, n=8 and 8 for Ctrl IgG and α-OPN antibody treatment group, respectively; */§/†P<0.05, §§/††P<0.01, §§§/†††P<0.001, §§§§P<0.0001 and not significant (ns) P>0.05. * indicates two-tailed, unpaired t-test, with Welch's correction when variances were significantly different based on F-test, comparing the two treatment groups for the same region, § indicates two-tailed, paired t-test comparison of peri-infarct to contralateral regions within the same treatment group/animal, and † indicates two-tailed, paired t-test comparison of core to contralateral regions within the same treatment group/animal. Scale bars: 50 μm in overlays and 10 μm in insets (**a-d**).

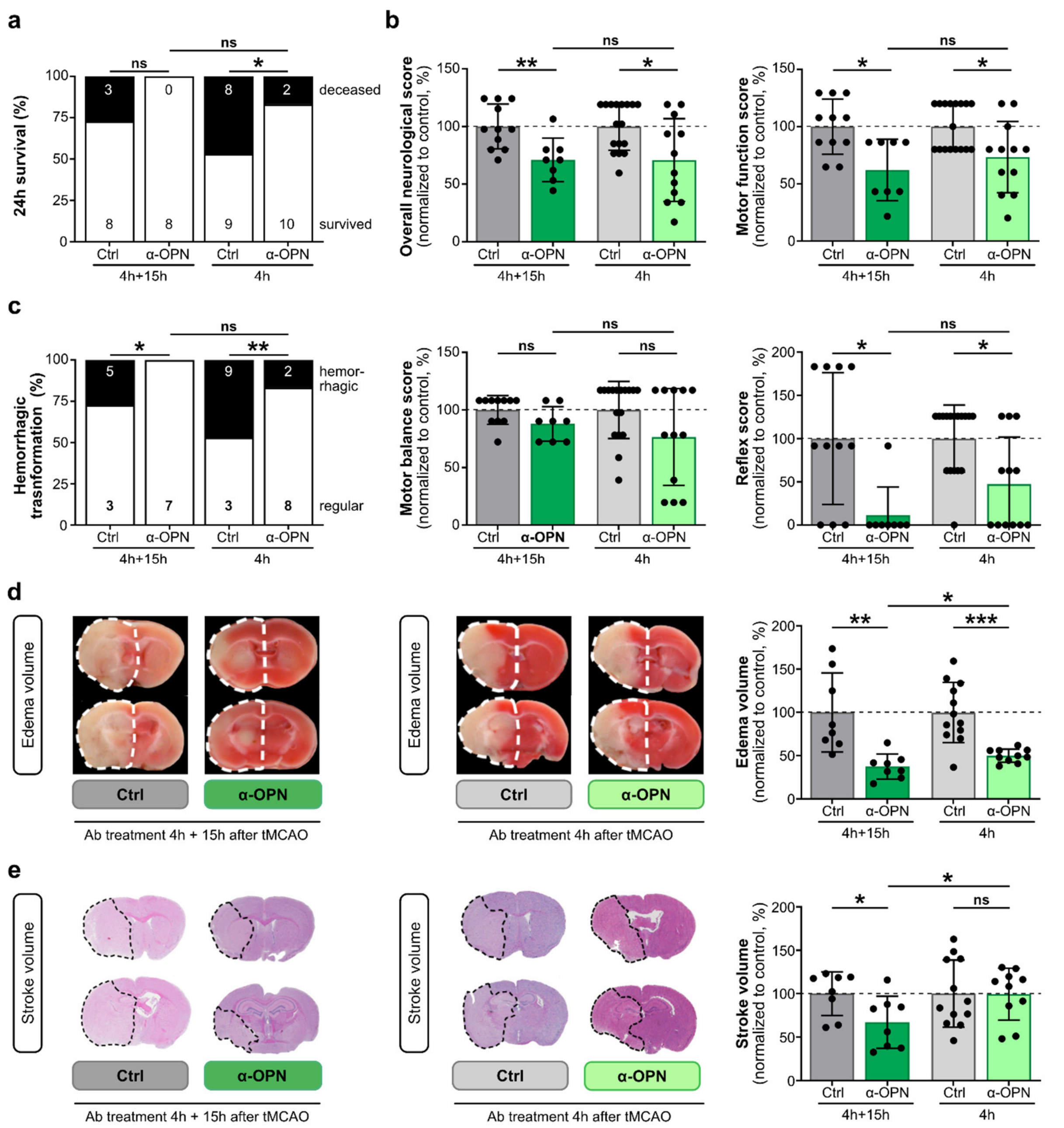

Supplementary Figure 10. Comparison of therapeutic effects of single and combined subcutaneous anti-osteopontin antibody administration on clinical outcome in mice post-acute ischemic stroke. a-e, Outcome parameters assessed 24 hours post-ischemic stroke in mice treated with Ctrl IgG or α-OPN antibody 4 hours (single administration in the early acute phase) or 4 hours and 15 hours (combined administration in the early and late acute phase) post-tMCAO. All animals that passed the exclusion criteria were included for the survival and neurological score analysis. For analysis of hemorrhagic transformation, edema and stroke volumes only mice that survived 24 hours were included. **a** and **b,** 24 hour survival proportion with numbers in histograms indicating animals that died or survived in each group (**a**), and total mNSS including motor balance, motor function and reflexes score (**b**), n=11 (Ctrl) and n=8 (α-

OPN) animals receiving combined treatment in the early and late acute phase, and n=17 (Ctrl) and n=12 (α-OPN) animals receiving single treatment in the early acute phase; *P<0.05 and not significant (ns) P>0.05 by Chi-square test for **A**, and *P<0.05, **P<0.01, and not significant (ns) P>0.05 by Mann Whitney test for **b. c**, Frequency of hemorrhagic transformation of stroke lesions. **d,** Representative TTC-stained coronal brain slices of mice injected subcutaneously with Ctrl IgG or α-OPN antibody 4 hours and 15 hours (combined administration in the early and late acute phase, left panel) or 4 hours (single administration in the early acute phase, middle panel) after tMCAO, demonstrating edema-induced expansion of ischemic hemispheres as indicated by white dotted line, and corresponding edema volumes (right panel). **e,** Representative H&E-stained coronal brain slices of mice injected subcutaneously with Ctrl IgG or α-OPN antibody 4 hours and 15 hours (combined administration in the early and late acute phase, left panel) or 4 hours (single administration in the early acute phase, middle panel) after tMCAO, demonstrating extent of infarction as indicated by black dotted line, and corresponding stroke volumes (right panel). For comparison of therapeutic effects of single and combined subcutaneous anti-osteopontin antibody administration (4h+15h vs. 4h) on clinical outcome in mice post-ischemic stroke neurological scores, edema and stroke volumes were normalized to controls in the single and combined treatment group as indicated by dashed line. n=8 and 8 for Ctrl IgG and α-OPN-treated animals receiving combined treatment in the early and late acute phase, and n=12 (Ctrl) and n=10 (α-OPN) mice receiving single treatment in the early acute phase; *P<0.05, **P<0.01, ***P<0.001 and not significant (ns) P>0.05 by Chi-square test for **C**, and two-tailed, unpaired t-test for **d** and **e**.

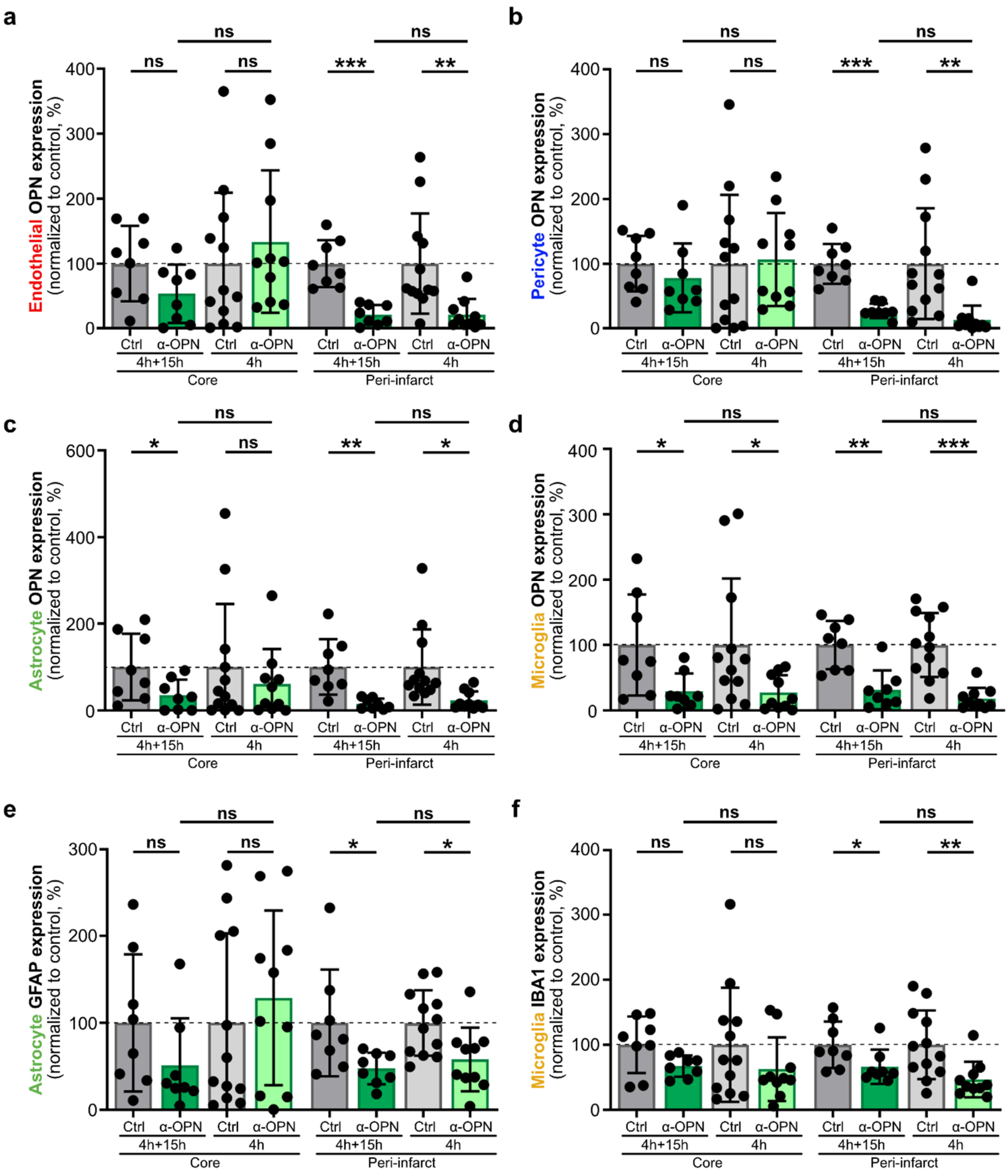

Supplementary Figure 11. Comparison of therapeutic effects of single and combined subcutaneous anti-osteopontin antibody administration on osteopontin expression in neurovascular unit cells in the infarct core and peri-infartc region post-acute ischemic stroke. a-d, Quantification of osteopontin (OPN) expression in core and peri-infarct endothelial cells (**a**), pericytes (**b**), astrocytes (**c**) and microglia/macrophages (**d**) 24 hours post-ischemic stroke in mice treated with Ctrl IgG or α-OPN antibody 4 hours and 15 hours (combined administration in the early and late acute phase) or 4 hours (single administration in the early acute phase) post-tMCAO. **e** and **f**, Quantification of GFAP expression in astrocytes (**e**) and IBA1 expression in microglia/macrophages (**f**) 24 hours post-ischemic stroke in mice treated

with Ctrl IgG or α-OPN antibody 4 hours and 15 hours (combined administration in the early and late acute phase) or 4 hours (single administration in the early acute phase) post-tMCAO. For comparison of therapeutic effects of single and combined subcutaneous anti-osteopontin antibody administration on OPN expression in dysregulated neurovascular unit cells in mice post-ischemic stroke, OPN expression intensity values were normalized to controls in the single and combined treatment group as indicated by dashed line. Three images per region and per animal were used, n=8 (Ctrl) and n=8 (α-OPN) animals receiving combined treatment in the early and late acute phase, and n=12 (Ctrl) and n=10 (α-OPN) animals receiving single treatment in the early acute phase; *P<0.05, **P<0.01, ***P<0.001 and not significant (ns) P>0.05 by two-tailed, unpaired t-test with Welch's correction when variances were significantly different based on F-test.

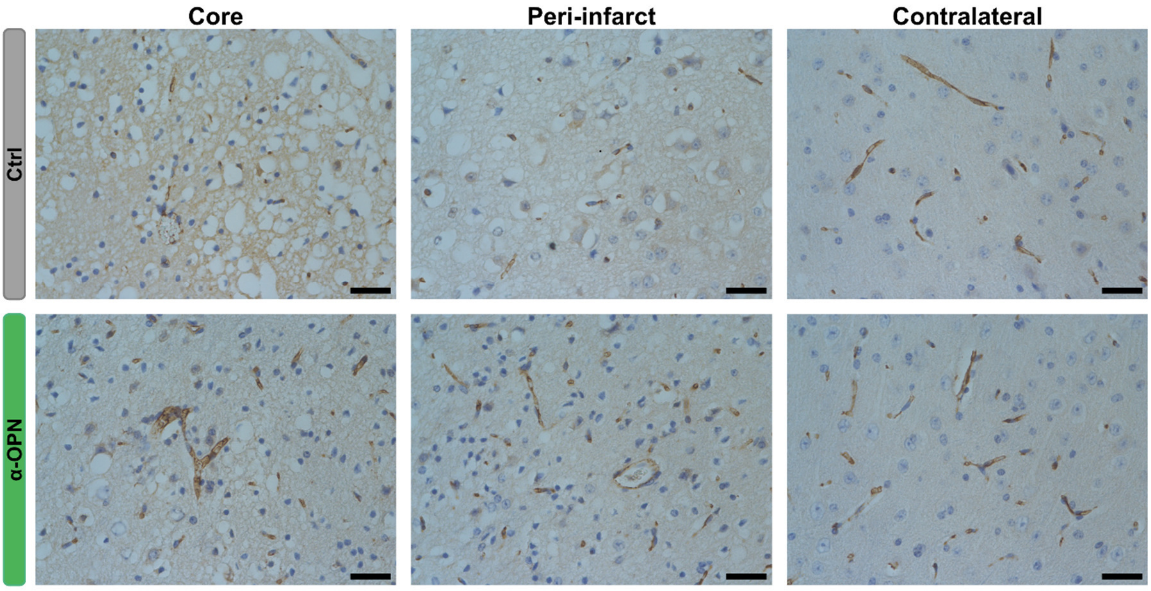

Supplementary Figure 12. Effect of anti-osteopontin antibody treatment on trans-blood-brain barrier albumin leakage in infarct core, peri-infarct region and contralateral hemisphere. Representative images of immunohistochemical staining for permeability marker albumin (brown) in the core, peri-infarct and contralateral regions of Ctrl IgG and α-OPN antibody-treated mice (n=3). Reduced albumin extravasation can be observed in the peri-infarct and infract core regions of the α-OPN antibody-treated animals compared to the Ctrl IgG-treated animals. These results support the IgG and fibrinogen permeability data.

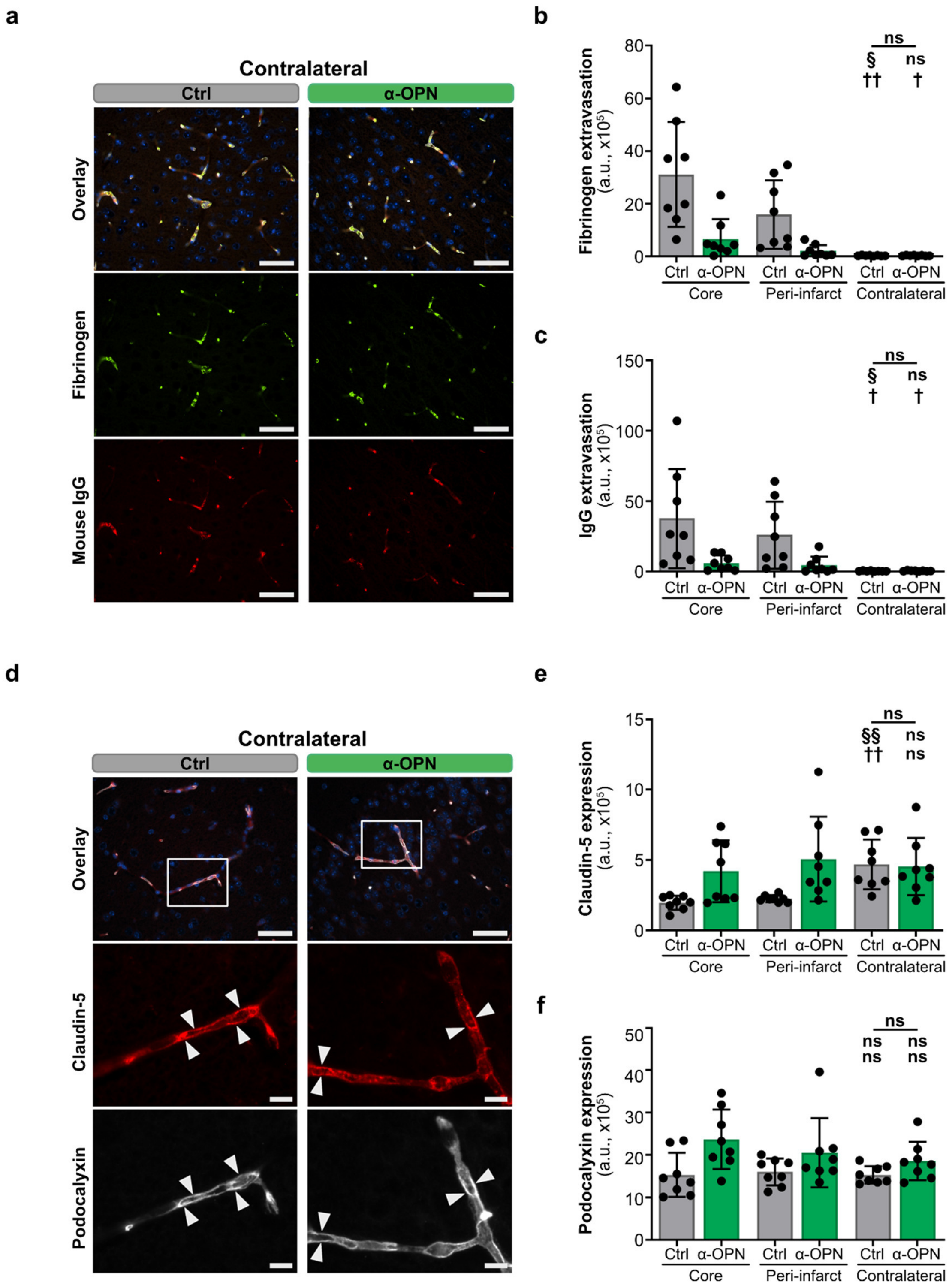

Supplementary Figure 13. Effect of anti-osteopontin antibody treatment on blood-brain barrier function in contralateral hemisphere, infarct core and peri-infarct region. a-c, Representative images of immunofluorescence staining for fibrinogen (green) and mouse immunoglobulin (IgG, red) in the contralateral hemisphere (**a**), and quantification for fibrinogen (**b**) and IgG (**c**) extravasation in the infarct core, peri-infarct region and contralateral hemisphere of Ctrl IgG and α-OPN antibody-treated mice. Podocalyxin (white) was used as

vessel marker as shown in overlay pictures. **d-f,** Representative images of immunofluorescence staining for endothelial tight junction protein claudin-5 (red, inset) and glycocalyx protein podocalyxin (white, inset) in the contralateral hemisphere (**d**), and quantification for claudin-5 (**e**) and podocalyxin expression (**f**) in the infarct core, peri-infarct region and contralateral hemisphere of Ctrl IgG and α-OPN antibody-treated mice. White arrowheads indicate claudin-5 and podocalyxin expression in brain microvascular endothelial cells in the contralateral hemisphere. For quantification three images/region/animal were utilized, n=8 and 8 for Ctrl IgG and α-OPN antibody treatment group, respectively; §/†$P<0.05$, §§/††$P<0.01$ and not significant (ns) $P>0.05$. Two-tailed, unpaired t-test comparing the two treatment groups for the contralateral hemisphere, with Welch's correction when variances were significantly different based on F-test. § indicates two-tailed, paired t-test comparison of peri-infarct to contralateral regions within the same treatment group/animal, and † indicates two-tailed, paired t-test comparison of core to contralateral regions within the same treatment group/animal. Scale bars: 50 µm in overlays and 10 µm in insets (**a** and **d**).

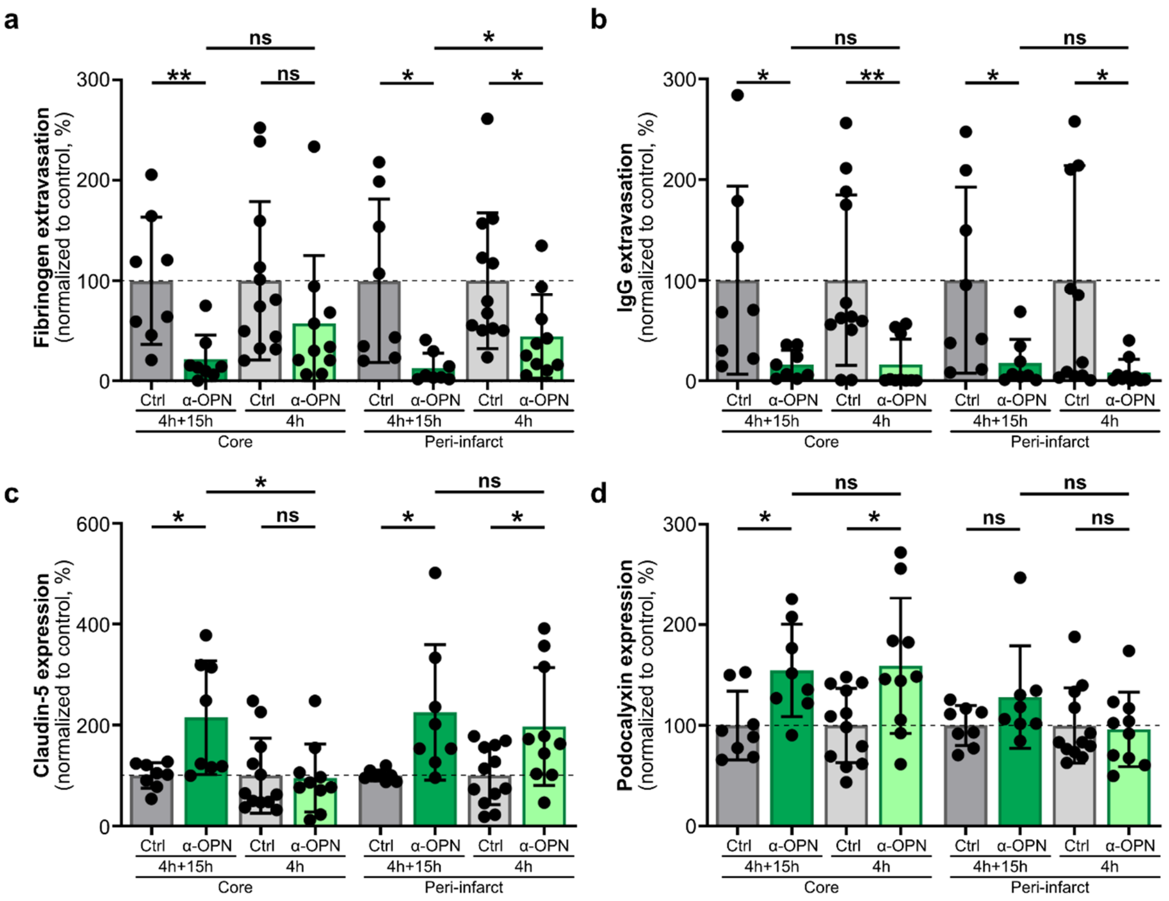

Supplementary Figure 14. Comparison of therapeutic effects of single and combined subcutaneous anti-osteopontin antibody administration on blood-brain barrier function in infarct core and peri-infarct region post-acute ischemic stroke. a and **b,** Quantification of fibrinogen (**a**) and mouse immunoglobulin (IgG, **b**) extravasation in infarct core and peri-infarct region 24 hours post-ischemic stroke in mice treated with Ctrl IgG or α-OPN antibody 4 hours and 15 hours (combined administration in the early and late acute phase) or 4 hours (single administration in the early acute phase) post-tMCAO. **c** and **d**, Quantification of microvascular claudin-5 (**c**) and podocalyxin expression (**d**) in infarct core and peri-infarct region 24 hours post-ischemic stroke in mice treated with Ctrl IgG or α-OPN antibody 4 hours and 15 hours (combined administration in the early and late acute phase) or 4 hours (single administration in the early acute phase) post-tMCAO. For comparison of therapeutic effects of single and combined subcutaneous anti-osteopontin antibody administration on fibrinogen and IgG extravasation, and microvascular claudin-5 and podocalyxin expression values were normalized to controls in the single and combined treatment group as indicated by dashed line. Three images per region and per animal were used, n=8 animals receiving combined treatment in the early and late acute phase, and n=12 (Ctrl) and n=10 (α-OPN) animals receiving single treatment in the early acute phase; *P<0.05, **P<0.01 and not significant (ns) P>0.05 by two-

tailed, unpaired t-test with Welch's correction when variances were significantly different based on F-test.

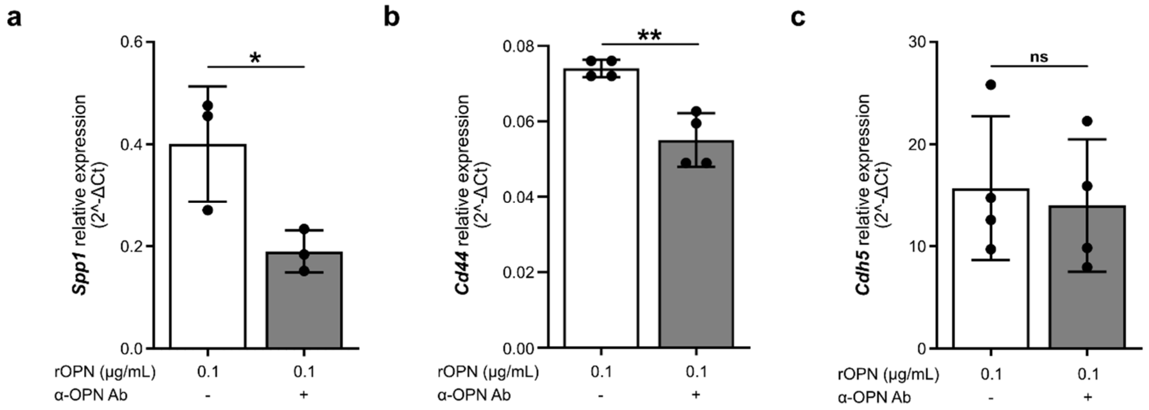

Supplementary Figure 15. Activation of osteopontin signaling and its response to anti-osteopontin antibody treatment in vitro. Activation of osteopontin (OPN) signaling by murine recombinant OPN (rOPN, 0.1 µg/mL) for 24 h and its inhibition by α-OPN antibody (0.1 µg/mL) was assessed at gene level by qRT-PCR on mouse brain microvascular endothelial cells in vitro for secreted phosphoprotein 1 (*Spp1*, encoding OPN, **a**) and its signaling pathway member cluster of differentiation 44 (*Cd44*, encoding CD44 receptor, **b**), and endothelial cadherin 5 (*Cdh5*, encoding vascular adherens junction protein VE-cadherin, **c**); n = 3–4 independent experiments, *P<0.05, **P<0.01 and not significant (ns) P>0.05 by two-tailed, unpaired t-test.

Supplemental Tables

Supplementary Table 1. Modified neurological severity score (mNSS) criteria for neurological deficits in mice post-acute ischemic stroke.

14-points modified Neurological Severity Score (mNSS)
**Motor tests (motor function scores)**
Flexion: raising the mouse by the tail (normal=0; maximum=3)
1 Flexion of forelimb
1 Flexion of hindlimb
1 Head movement more than 10 ° to the vertical axis within 30 seconds
Gait: placing the mouse on the floor (normal=0; maximum=3)
0 Normal walk
1 Inability to walk straight
2 Circling towards the paretic side
3 Falling towards the paretic side
**Beam balance tests (motor balance scores;** normal=0; maximum=6)
0 Balances with steady posture
1 Grasps side of beam
2 Hugs the beam and one limb falls down from the beam
3 Hugs the beam and two limbs fall down from the beam, or spins on beam (>30 seconds)
4 Attempts to balance on the beam but falls off (>20 seconds)
5 Attempts to balance on the beam but falls off (>10 seconds)
6 Falls off; no attempt to balance or hang on to the beam (<10 seconds)
**Sensory function tests** (**reflexes scores;** normal=0; maximum=2)
1 Absence of corneal reflex
1 Absence of Pinna reflex

Supplementary Table 2. Antibody details for immunofluorescence staining.

Antibody (species)	Immunogen/epitope	Company	Reference	Concentration	Dilution
CD13 (Gt), poly	Amino acids 69-966	R&D systems	AF2335	0.2 mg/mL	1:200
CD44 (Rt), mono	Amino acids 145-186	BioLegend	103002	0.5 mg/mL	1:200
Claudin-5 (Ms), mono	N/A from the vendor	ThermoFisher Scientific	352500	0.5 mg/mL	1:200
Fibrinogen (Rb), poly	N/A from the vendor	LSBio	LS C150799	17mg/mL	1:200
GFAP (Gt), poly	N/A from the vendor	Abcam	ab53554	0.5 mg/mL	1:250
Iba1 (Ms), mono	N/A from the vendor	Merck	SAB2702364	2.03mg/mL	1:200
Osteopontin (Rb), poly	Amino acids 13-300	Proteintech	22952-1-AP	1.0 mg/mL	1:500
Podocalyxin (Gt), poly	Amino acids 21-402	R&D systems	AF1556	0.2 mg/mL	1:200
Dk anti-Gt 650, poly	N/A from the vendor	ThermoFisher Scientific	SA5-10089	0.5 mg/mL	1:200
Dk anti-Rb 550, poly	N/A from the vendor	ThermoFisher Scientific	SA5-10039	0.5 mg/mL	1:200
Dk anti-Rb 488, poly	N/A from the vendor	ThermoFisher Scientific	SA5-10038	0.5 mg/mL	1:200
Dk anti-Ms 650, poly	N/A from the vendor	ThermoFisher Scientific	SA5-10169	0.5 mg/mL	1:200
Dk anti-Ms 550, poly	N/A from the vendor	ThermoFisher Scientific	SA5-10167	0.5 mg/mL	1:200
Dk anti-Rt 488, poly	N/A from the vendor	ThermoFisher Scientific	SA5-10026	0.5 mg/mL	1:200

Gt anti-Rt 568, poly	N/A from the vendor	ThermoFisher Scientific	A11077	0.5 mg/mL	1:200

Species: Rb, rabbit; Gt, goat; Ms, mouse; Rt, rat; Dk, donkey; mono, monoclonal; poly, polyclonal.

Supplementary Table 3. Sequences of the primer pairs used for quantitative real-time PCR.

**Gene name**	**Forward (5'-3')**	**Reverse (5'-3')**
Rplp0	gtgtttgacaacggcagcatt	tctccacagacaatgccagga
Cd44	gctacagcaagaagggcgagt	cctgatctccagtaggctgttc
Cdh5	gcccagccctacgaacctaaa	gggtgaagttgctgtcctcgt
Spp1	ccaatgaaagccatgaccaca	atccgactgatcggcactctc
